# Post-discharge opioid prescribing after surgery in the United States: a population-based analysis of specialty variation and prescribing intensity

**DOI:** 10.1016/j.lana.2026.101456

**Published:** 2026-03-13

**Authors:** Adriana C. Panayi, Dany Y. Matar, Thomas Schaschinger, Tobias Niederegger, Jule Brandt, Iman Ghanad, Dennis P. Orgill, Gabriel Hundeshagen

**Affiliations:** a Department of Cranio-Maxillofacial and Oral Surgery, University Hospital Zurich, University of Zurich, Rämistrasse 100, Zurich, 8091, Switzerland; b Department of Plastic and Reconstructive Surgery, Johns Hopkins Hospital, Johns Hopkins University School of Medicine, Baltimore, MD, USA; c Medical Faculty, University of Heidelberg, Heidelberg, Germany; d Department of Oral and Maxillofacial Surgery, Charité – Universitätsmedizin Berlin, Corporate Member of Freie Universität Berlin, Humboldt-Universität zu Berlin, Berlin, Germany; e Division of Plastic Surgery, Department of Surgery, Brigham and Women's Hospital, Harvard Medical School, Boston, MA, USA; f BG Klinik Ludwigshafen, Department of Hand, Plastic, and Reconstructive Surgery, Burn Center at Heidelberg University, Ludwig-Guttmann-Str. 13, 67071, Ludwigshafen, Germany

**Keywords:** Quality improvement, Surgery, Opioids, Prognostic, Risk factor, Critical care

## Abstract

**Background:**

The transition from hospital to home after surgery is a vulnerable period, yet post-discharge opioid prescribing varies widely across surgical specialties. This study aimed to characterize these prescribing patterns and evaluate their implications for early postoperative outcomes.

**Methods:**

We performed a retrospective cohort study using the 2024 American College of Surgeons National Surgical Quality Improvement Program (ACS-NSQIP) database. Adult surgical patients who survived to discharge and had complete discharge analgesic data were included. Opioid prescribing was characterized by daily morphine milligram equivalents (MME), cumulative dose, duration, route, dosing frequency, and renewals. Multivariable regression adjusted for demographics, comorbidities, specialty, operative characteristics, and outcomes.

**Findings:**

Among 945,505 surgical patients, 683,828 (72.3%) were discharged with an opioid prescription. Prescribing varied by specialty and procedure, with a mean daily dose of 44.8 MME (Standard deviation, SD 122.1), mean duration of 4.2 days (SD 3.3), and prescription renewals in 28,385 (4.2%) patients. Patients discharged with opioids had shorter hospital stays (2.0 vs 3.3 days; p < 0.001) and lower rates of complications (7.7% vs 11.0%; p < 0.0001), reflecting preferential prescribing among clinically stable patients. Surgical specialty and anesthesia type were the strongest predictors of prescribing intensity, with higher odds of high-intensity prescribing following orthopedic (adjusted Odds Ratio, aOR 6.79, 95% Confidence Interval, CI 6.64–6.93) and neurosurgical procedures (aOR 5.66, CI 5.50–5.83), and spinal anesthesia (aOR 2.27, CI 2.21–2.33; all p < 0.001).

**Interpretation:**

Despite national efforts to reduce opioid use, most surgical patients continue to receive opioids at discharge, with specialty-specific variation. Differences in early postoperative outcomes should be interpreted as markers of clinical selection and recovery trajectory rather than evidence of opioid-related benefit. Procedure-specific, recovery-informed prescribing guidelines are needed to minimize avoidable opioid prescribing while ensuring adequate analgesia.

**Funding:**

None.

**Research in context**

**Evidence before this study**

Using the search terms (“post-discharge opioid∗” OR “postoperative opioid∗”) AND (“surgery” OR “surgical procedure∗”) AND (“morphine milligram equivalents” OR “MME”) AND (“outcomes” OR “readmission” OR “delirium”), we searched PubMed for studies published up to January 2024 that examined opioid prescribing at hospital discharge after surgery. Prior studies consistently demonstrated wide variation in discharge opioid prescribing and frequent prescribing of quantities exceeding patient-reported needs or guideline recommendations. This literature includes both single-center and multi-institutional studies, as well as analyses using administrative claims data and specialty-specific cohorts. However, much of the existing evidence is limited to selected procedures, individual surgical specialties, or claims-based outcomes, constraining direct cross-specialty comparison within a single harmonized clinical dataset. Evidence linking post-discharge opioid prescribing to early postoperative outcomes, such as 30-day complications, readmissions, or postoperative delirium, has been inconsistent and is often difficult to interpret due to confounding by patient acuity, recovery trajectory, and clinical selection.

**Added value of this study**

Using a national cohort of 945,505 adult surgical patients from the 2024 American College of Surgeons National Surgical Quality Improvement Program (ACS-NSQIP), the first registry release to include detailed post-discharge opioid prescribing variables, we comprehensively characterized post-discharge opioid prescribing across surgical specialties and procedures. Opioid exposure was quantified using standardized measures of daily MME, cumulative dose, duration, formulation, and renewals, allowing prescribing intensity to be assessed as a multidimensional construct rather than a single metric. By adjusting for patient demographics, comorbidities, functional status, operative characteristics, anesthesia type, and postoperative clinical course, we identified surgical specialty and recovery trajectory as the strongest predictors of prescribing intensity. By explicitly contextualizing prescribing patterns alongside markers of postoperative stability, we demonstrate that lower complication rates and shorter hospital stays among opioid-prescribed patients reflect selective prescribing in clinically stable individuals rather than a causal benefit of opioid exposure.

**Implications of all the available evidence**

Taken together, available evidence indicates that post-discharge opioid prescribing in the United States remains common and frequently exceeds recommended quantities, with persistent and substantial variation across surgical specialties and procedures. Our findings reinforce that apparent associations between opioid prescribing and favorable early postoperative outcomes largely reflect patient selection and recovery status, not opioid-related benefit. In this context, further reductions in opioid exposure are unlikely to be achieved through uniform prescribing targets alone. Instead, procedure-specific, recovery-informed prescribing frameworks, grounded in contemporary benchmarking and attentive to functional and neurologic vulnerability, may better balance adequate pain control with minimization of unnecessary opioid exposure. Such approaches align stewardship efforts with clinical reality and may improve patient safety during the vulnerable transition from hospital to home.

## Introduction

Postoperative pain management remains a central component of surgical care, and opioid medications continue to function as the primary analgesic strategy for most surgical patients in the United States (US).^1^ However, patterns of post-discharge opioid prescribing have come under scrutiny as concerns around overprescription, prolonged use, and opioid-related harms have intensified. The transition from inpatient to outpatient recovery represents a particularly vulnerable period during which patients may experience heightened risks of misuse, persistent opioid use, emergency department visits, and other preventable complications.^2^, ^3^, ^4^

Despite substantial national attention to the opioid epidemic, wide variation persists in prescribing practices across specialties, patient populations, and healthcare systems.^5^, ^6^, ^7^, ^8^, ^9^, ^10^, ^11^, ^12^, ^13^ Seminal work by Brummett and colleagues established that new persistent opioid use represents a common but underappreciated surgical complication, affecting 3–10% of previously opioid-naive patients across both minor and major procedures.^14^ Subsequent analyses have sought to define optimal prescription duration, with procedure-specific data suggesting that the lowest rates of refills occur at initial prescription lengths of 4–9 days for general surgery and 6–15 days for musculoskeletal procedures.^15^ Work in trauma populations has further demonstrated that lower socioeconomic status and higher injury severity are independent predictors of sustained postoperative opioid use.^16^ More recently, Schoenfeld and colleagues conducted one of the largest longitudinal assessments of postoperative opioid use in the US, demonstrating clinically meaningful reductions in long-term prescription opioid use following surgery between 2017–2019 and 2020–2022 across all census divisions, racial groups, and socioeconomic strata. These findings were interpreted as reflecting the delayed but cumulative impact of Centers for Disease Control and Prevention (CDC) and Veterans Affairs/Department of Defense (VA/DoD) practice guidelines.^17^ Post-discharge prescribing remains less standardized and continues to vary widely across practice settings. Less is known about how contemporary discharge prescribing patterns relate to validated 30-day postoperative outcomes within a unified, cross-specialty analytic framework.^18^^,^^19^

Prescribing practices and regulatory environments have evolved substantially in recent years. Many surgical patients, particularly those with limited prior opioid exposure, multiple comorbidities, or psychosocial vulnerability, may be disproportionately susceptible to opioid-related adverse events in the immediate post-discharge period.^20^ In the current post-stewardship era, perioperative protocols still lack widely adopted, evidence-based benchmarks for discharge dose, duration, and regimen complexity, contributing to persistent variability at the time of discharge.^21^, ^22^, ^23^ Critically, existing work has been largely limited to opioid-naïve populations, often excluding patients with preoperative exposure, frailty, or cognitive impairment, and has rarely examined validated 30-day surgical outcomes across the full spectrum of surgical specialties within a single unified framework.

The recent availability of post-discharge opioid prescribing variables within the ACS-NSQIP provides an opportunity to re-examine this issue using a clinically abstracted national registry with validated 30-day outcomes. The NSQIP enables consistent risk adjustment, procedure-level granularity, and direct cross-specialty comparison within a harmonized cohort. Importantly, the 2024 dataset reflects prescribing behavior in a mature, post-pandemic, post-stewardship environment, allowing contemporary benchmarking rather than reassessment of earlier phases of opioid expansion.^24^^,^^25^

We conducted a retrospective cohort study using the 2024 ACS-NSQIP dataset to: (1) benchmark contemporary post-discharge opioid prescribing intensity using multiple complementary measures, including dose, duration, renewals, and regimen complexity; (2) examine associations between prescribing intensity and three principal short-term outcomes (unplanned 30-day readmission, major postoperative complications, and postoperative delirium) while explicitly framing discharge prescribing as a marker of postoperative recovery trajectory rather than a causal exposure; (3) determine whether these relationships differed across vulnerable subgroups, including older adults, persons with cognitive impairment, those with frailty indicators, and individuals undergoing urgent or emergent operations.

## Methods

### Data source

The ACS-NSQIP is a quality improvement program that collects detailed clinical information from participating hospitals to monitor and improve surgical outcomes. Trained reviewers abstract patient-level data from medical records using standardized protocols, and data integrity is maintained through routine peer review and random auditing. All information is fully de-identified. ACS-NSQIP includes a large, diverse sample of US hospitals spanning academic and community settings, with standardized data abstraction and risk adjustment. However, participation is voluntary, and the cohort may overrepresent larger and higher-resourced institutions, which should be considered when generalizing findings to all US surgical practice. This study used the 2024 ACS-NSQIP dataset, the first release to include detailed post-discharge opioid prescribing variables linked to clinically abstracted perioperative data and validated 30-day outcomes.

### Ethical approval and informed consent

This study was conducted with institutional review board (IRB) approval from Brigham and Women's Hospital, Boston, MA, USA (protocol 2013P001244). As this study used a fully de-identified, retrospective administrative dataset, individual patient consent was not required. This study was reported in accordance with the Strengthening the Reporting of Observational Studies in Epidemiology (STROBE) guidelines and the REporting of studies Conducted using Observational Routinely-collected Data (RECORD) extension.^26^^,^^27^

### Patient selection

**Fig. 1:**
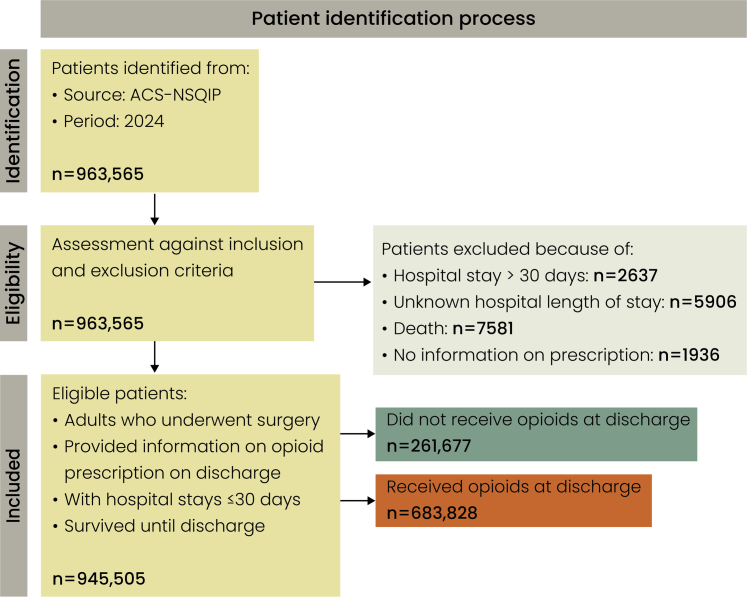
**Patient identification process and cohort grouping.** A total of 963,565 patients were identified from the 2024 ACS-NSQIP dataset. Patients were excluded for hospital length of stay >30 days (n = 2637), unknown length of stay (n = 5906), death prior to discharge (n = 7581), or missing opioid prescription information (n = 1936). After applying inclusion and exclusion criteria, 945,505 adults who underwent surgery, survived to discharge, and had complete discharge opioid data were included. Of these, 261,677 patients did not receive opioids at discharge, while 683,828 received an opioid prescription at discharge.

Only patients with complete documentation of post-discharge opioid prescribing were included. Individuals with missing opioid data or hospital stays exceeding 30 days were excluded (Fig. 1). Because the ACS-NSQIP captures outcomes only within the first 30 postoperative days, patients with longer lengths of stay have incomplete or non-comparable post-discharge outcome data. These exclusions ensured a clinically comparable cohort in which discharge prescribing reflected routine postoperative recovery rather than prolonged inpatient complexity.

### Variable extraction

Preoperative and intraoperative variables (Table 1) were extracted, including demographics (age, self-designated gender, race and ethnicity), comorbidities, functional status, laboratory values, operative characteristics, case type, surgical specialty, anesthesia type, and care setting. Procedures were identified using 21 possible Current Procedural Terminology (CPT) code fields and were subsequently categorized by anatomic region and surgical specialty (Supplementary Table S1).

**Table 1:** Preoperative and surgical characteristics of patients discharged with and without opioids.

Characteristic	Total patients (n = 945,505)	No opioids (n = 261,677)	Opioids (n = 683,828)	p-value	Cramér's V
Age, mean years (SD)	57 (17)	58 (17)	56 (17)	**<0.0001**	0.15
BMI, mean kg/m^2^ (SD)	30 (7.1)	30 (7.1)	31 (7.1)	**<0.0001**	<0.001
Self-designated gender				**<0.0001**	0.02
Female	549,590 (58)	148,103 (57)	401,487 (59)		
Male	395,574 (42)	113,500 (43)	282,074 (41)		
Non-binary	337 (0.0)	72 (0.0)	265 (0.0)		
Intersex	4 (0.0)	2 (0.0)	2 (0.0)		
Self-designated race				**<0.0001**	0.08
Black or African American	93,848 (9.9)	23,773 (9.1)	70,075 (10)		
White	567,393 (60)	137,485 (53)	429,908 (63)		
Unknown	227,177 (24)	81,753 (31)	145,424 (21)		
American Indian or Alaska Native	6985 (0.7)	1188 (0.5)	5797 (0.9)		
Multiple Races	6593 (0.7)	2207 (0.8)	4386 (0.6)		
Asian	36,394 (3.8)	13,115 (5.0)	23,279 (3.4)		
Hispanic or Latino	2121 (0.2)	513 (0.2)	1608 (0.2)		
Native Hawaiian or Other Pacific Islander	3835 (0.4)	779 (0.3)	3056 (0.5)		
Middle Eastern or North African	1159 (0.1)	864 (0.3)	295 (0.0)		
Self-Designated Hispanic Ethnicity	104,617 (11)	27,849 (11)	76,768 (11)	**<0.0001**	0.01
Comorbidities					
Diabetes				**<0.0001**	0.04
No	792,290 (84)	214,524 (82)	577,766 (84)		
Non-insulin	108,789 (12)	31,768 (12)	77,021 (11)		
Insulin	44,426 (4.7)	15,385 (5.9)	29,041 (4.2)		
Current Smoker	111,158 (12)	31,671 (12)	79,487 (12)	**<0.0001**	0.01
Ventilator	808 (0.085)	518 (0.2)	290 (0.0)	**<0.0001**	0.02
COPD	33,682 (3.6)	11,320 (4.3)	22,362 (3.3)	**<0.0001**	0.03
Ascites	4194 (0.4)	1728 (0.7)	2466 (0.4)	**<0.0001**	0.02
CHF	33,165 (3.5)	12,516 (4.8)	20,649 (3.0)	**<0.0001**	0.04
Hypertension	403,903 (43)	116,005 (44)	287,898 (42)	**<0.0001**	0.02
Renal Failure	1176 (0.1)	593 (0.23)	583 (0.1)	**<0.0001**	0.02
Dialysis	6277 (0.7)	2944 (1.1)	3333 (0.5)	**<0.0001**	0.04
Disseminated Cancer	19,162 (2.0)	6457 (2.5)	12,705 (1.9)	**<0.0001**	0.02
Corticosteroid use	42,280 (4.5)	11,818 (4.5)	30,462 (4.5)	0.20	0.001
Bleeding Disorder	30,654 (3.2)	12,314 (4.7)	18,340 (2.7)	**<0.0001**	0.05
Blood Transfusion	5690 (0.6)	2700 (1.0)	2990 (0.4)	**<0.0001**	0.03
Sepsis	55,254 (5.8)	19,817 (7.6)	35,437 (5.2)	**<0.0001**	0.046
Fall in past 6 months	25,474 (2.7)	10,084 (3.9)	15,390 (2.3)	**<0.0001**	0.03
Dementia	12,480 (1.3)	6173 (2.4)	6307 (0.9)	**<0.0001**	0.08
Home support				0.05	0.01
Unknown	11,821 (1.3)	4341 (1.7)	7480 (1.1)		
Lives at home with others	99,728 (11)	35,708 (14)	64,020 (9.4)		
Lives alone at home	33,335 (3.5)	11,738 (4.5)	21,597 (3.2)		
Functional status				**<0.0001**	0.07
Independent	906,800 (96)	247,148 (94)	659,652 (96)		
Partially dependent	16,458 (1.7)	7677 (2.9)	8781 (1.3)		
Totally dependent	2309 (0.2)	1358 (0.5)	951 (0.1)		
Unknown	19,938 (2.1)	5494 (2.1)	14,444 (2.1)		
Blood lab values					
Sodium, mean mEq/L (SD)	139 (2.9)	139 (3.1)	139 (2.8)	**<0.0001**	<0.001
BUN, mean mg/dL (SD)	16 (9.0)	17 (11)	16 (8.2)	**<0.0001**	0.12
Creatinine, mean mg/dL (SD)	1.0 (0.7)	1.0 (0.8)	0.9 (0.6)	**<0.0001**	0.10
Albumin, mean g/dL (SD)	4.0 (0.6)	3.9 (0.6)	4.1 (0.5)	**<0.0001**	<0.001
WBC count, mean × 10^3^/μL (SD)	8.1 (3.5)	8.3 (3.8)	8.0 (3.4)	**<0.0001**	0.10
Hematocrit, mean % (SD)	40 (5.2)	39 (5.5)	40 (5.0)	**<0.0001**	<0.001
**Surgical Characteristic**					

Operation time, mean minutes (SD)	117 (95)	114 (99)	118 (94)	**<0.0001**	<0.001
Case type				**<0.0001**	0.09
Elective	779,485 (82)	201,407 (77)	578,078 (85)		
Emergent	70,978 (7.5)	27,558 (11)	43,420 (6.3)		
Urgent	95,042 (10)	32,712 (13)	62,330 (9.1)		
Surgical specialty				**<0.0001**	0.21
Plastics	30,903 (3.3)	9390 (3.6)	21,513 (3.1)		
Gynecology	98,602 (10)	24,938 (9.5)	73,664 (11)		
General Surgery	390,827 (41)	115,367 (44)	275,460 (40)		
Otolaryngology	23,203 (2.5)	8343 (3.2)	14,860 (2.2)		
Urology	63,306 (6.7)	28,649 (11)	34,657 (5.1)		
Obstetrics	19,032 (2.0)	6324 (2.4)	12,708 (1.9)		
Neurosurgery	48,386 (5.1)	11,227 (4.3)	37,159 (5.4)		
Vascular	27,107 (2.9)	15,369 (5.9)	11,738 (1.7)		
Thoracic	12,754 (1.3)	2816 (1.1)	9938 (1.5)		
Orthopedics	227,811 (24)	36,905 (14)	190,906 (28)		
Cardiac surgery	3511 (0.4)	2301 (0.9)	1210 (0.2)		
Interventional radiology	63 (0.0)	48 (0.0)	15 (0.0)		
Anesthesia				**<0.0001**	0.07
General	819,094 (87)	232,845 (89)	586,249 (86)		
Epidural	5154 (0.6)	1341 (0.5)	3813 (0.6)		
MAC/IV sedation	50,970 (5.4)	14,255 (5.4)	36,715 (5.4)		
Regional	5904 (0.6)	1272 (0.5)	4632 (0.7)		
Spinal	62,985 (6.7)	11,086 (4.2)	51,899 (7.6)		
Local	953 (0.1)	658 (0.3)	295 (0.0)		
Unknown	403 (0.0)	204 (0.1)	199 (0.0)		
Setting				**<0.0001**	0.10
Outpatient	499,382 (53)	116,879 (45)	382,503 (56)		
Inpatient	446,123 (47)	144,798 (55)	301,325 (44)		

BMI, Body Mass Index; BUN, Blood Urea Nitrogen; CHF, Congestive Heart Failure; COPD, Chronic Obstructive Pulmonary Disease; MAC/IV, Monitored Anesthesia Care/Intravenous sedation; SD, Standard Deviation; WBC, White Blood Cell count.

Data are reported as n (%), unless otherwise specified. Cramér's V is provided to quantify the magnitude of association between opioid prescribing status and each outcome. Statistical significance was defined as p < 0.05.

Covariates were prespecified a priori and selected to represent established perioperative risk domains rather than to identify novel predictors. These domains included patient demographics, baseline health status, functional and social vulnerability, operative complexity, anesthesia approach, surgical specialty, and early postoperative clinical trajectory.

Thirty-day postoperative outcomes included all-cause and unplanned readmission, time from discharge to readmission, and major postoperative complications. Complications were defined as any of the following: superficial, deep incisional or organ space infection, dehiscence, bleeding/transfusion, pneumonia, reintubation, ventilator use >48 h, pulmonary embolism, renal insufficiency, dialysis, stroke, cardiac arrest, myocardial infarction, deep vein thrombosis, *C. difficile* colitis, sepsis, or septic shock. Postoperative delirium measures included delirium screening, tool-identified delirium, and clinically diagnosed delirium. Additional variables included discharge destination, need for home services, functional status at discharge, total length of stay (LOS), transfer to higher-level care, end-of-life or withdrawal-of-care decisions, and postoperative COVID-19 status (Supplementary Table S2).

Variables describing opioid prescribing at discharge (Table 2) included medication type, formulation (pill, liquid, patch, sublingual, or other), dose strength, dose units, volume for liquid formulations, dosing frequency, total doses dispensed, and any renewal or continuation after discharge. Up to five opioid prescriptions were available per patient, enabling detailed assessment of prescribing patterns. Prescribing intensity was conceptualized as a multidimensional construct encompassing dose, duration, and regimen complexity rather than a single summary metric. The primary exposure was receipt of any opioid prescription at discharge.

**Table 2:** Summary of postoperative opioid prescribing patterns among patients who received an opioid prescription at discharge.

Characteristic	Patients with available data among 683,828 patients with opioid prescriptions	Mean (SD) or n (%)
Total MME per day	471,389 (68.9%)	44.8 (122.1)
Total MME over period	392,582 (57.4%)	197.3 (785.5)
Total doses (all opioids)	415,431 (60.8%)	21.0 (20.4)
Maximum days supplied	399,093 (58.4%)	4.2 (3.3)
Route of administration	510,010 (74.6%), 510,424 total prescriptions	
Liquid		3545 (0.7%)
Patch		207 (0.0%)
Pill		505,546 (99%)
Sublingual/Lollipop form		388 (0.1%)
Other		738 (0.2%)
Dosing interval	492,390 (72.0%), 513,182 total prescriptions	
Every hour		58 (0.0%)
Every 2 h		1537 (0.3%)
Every 4 h		211,438 (41.1%)
Every 6 h		233,699 (45.5%)
Every 8 h		24,521 (4.8%)
Every 12 h		7037 (1.4%)
Every 24 h		1116 (0.2%)
Every 2–4 h		2093 (0.4%)
Every 4–6 h		19,996 (3.9%)
Every 6–8 h		2349 (0.5%)
Other		10,338 (2.0%)
Prescription renewal	683,828 (100.0%)	
Yes		28,385 (4.2%)
No		655,443 (95.8%)
Intensity	478,920 (70.0%)	
Short/low		287,672 (42.1%)
Intermediate		71,631 (10.5%)
High		119,617 (17.5%)
Type (not mutually exclusive)	683,828 (100.0%)	
Codeine		20,498 (3.0%)
Hydrocodone		86,731 (12.7%)
Hydromorphone		62,054 (9.1%)
Morphine		3633 (0.5%)
Oxycodone		427,945 (62.6%)
Tramadol		98,022 (14.3%)
Other		6886 (1.0%)

Measures include mean MME per day, total MME over the prescription period, total number of opioid doses dispensed, days supplied, and corresponding standard deviations (SD). Additional prescription characteristics, such as route of administration, dosing interval, renewal status, intensity category, and opioid medication type, are reported as n (%) of all patients receiving an opioid prescription.

### Calculation of MME

MME were calculated using standard oral conversion factors: daily MME = (dose per administration in mg × doses per day) × opioid-specific conversion factor.^28^ MMEs were calculated only for prescriptions with convertible dose units, dosing frequencies, and routes mapped to oral administration. For patients with multiple prescriptions, daily MMEs were summed. Days supplied were derived by dividing the total number of dispensed doses by estimated doses per day. Patients with at least one convertible prescription were included; those missing all convertible fields were classified as unknown.

### Assessment of missing data and cohort selection

In descriptive analyses, observations with missing values were excluded from statistical comparisons and effect-size calculations. For multivariable analyses, missing covariate values were imputed as part of the preprocessing pipeline to allow inclusion of all patients in adjusted models. To evaluate the potential impact of missing post-discharge opioid prescribing data, we compared baseline characteristics and postoperative outcomes between patients included in our analytic cohort and those excluded due to missing opioid data or prolonged hospitalization (>30 days). These comparisons were performed to assess selection effects rather than to infer outcome differences attributable to opioid exposure.

### Statistical analysis

Raw data were processed into analyzable files using SPSS Version 29 and stored in LabArchives, then analyzed using Python (Google Colab). Descriptive statistics compared characteristics by opioid prescribing status using χ^2^ tests for categorical variables and independent t-tests for continuous variables, with effect sizes expressed as Cramér's V and Cohen's d.

The distribution of continuous variables was formally assessed using D'Agostino's K-squared test. Although these tests indicated statistically significant deviations from normality (p < 0.05), this was expected given the very large sample size and reflects the high sensitivity of normality tests rather than clinically meaningful departures from normality. Accordingly, Welch's t-test was used, leveraging the robustness of the t-test under the Central Limit Theorem and allowing for unequal variances between groups. Continuous variables are therefore reported as mean (standard deviation) for clinical interpretability. Given the right-skewed distribution of post-discharge opioid prescribing, mean-based summaries were used to reflect aggregate population exposure, while skew was addressed analytically through prespecified prescribing intensity categories and specialty-stratified analyses.

To evaluate the factors associated with postoperative opioid prescribing, multivariable logistic regression models were constructed using a Ridge-penalized (L2) framework. Ridge regularization was selected a priori to stabilize estimates in a high-dimensional setting and to prioritize overall domain-level effects over individual covariate discovery. The primary outcomes evaluated included the likelihood of receiving any opioid prescription at discharge, as well as prescribing intensity categorized by daily MME: low/short intensity (<30 MME/day), intermediate intensity (30–45 MME/day), and high intensity (>45 MME/day), using no-opioid patients as the reference group. Data preprocessing included a stratified imputation approach where numeric variables were adjusted via mean imputation and categorical variables via mode imputation. Continuous features were normalized using Z-score standardization to ensure a uniform scale. Categorical data were transformed into binary indicators through one-hot encoding, incorporating automated reference category identification to avoid the dummy variable trap.

For final inferential analyses, zero-variance features were removed and multicollinearity addressed through full-rank feature selection before fitting an unpenalized maximum likelihood logistic regression model to estimate the OR with 95% CI. All multivariable models included a prespecified set of covariates selected a priori based on clinical relevance and data availability within the NSQIP, rather than data-driven variable selection. These included demographic variables, anthropometric measures, preoperative comorbidities, preoperative laboratory values, functional and social factors, operative characteristics, anesthesia type, surgical specialty, and postoperative clinical trajectory variables. All covariates included in the final models are displayed in Table 3. Sample sizes for individual prescription characteristics vary slightly due to incomplete population of specific discharge opioid fields within ACS-NSQIP; all analyses were otherwise conducted within a consistent analytic cohort. Statistical significance was defined as p < 0.05 for all analyses.

**Table 3:** Multivariable-Adjusted Associations for Opioid Prescription Intensity and Any Opioid prescription.

Am. Indian/Alaska Nat., American Indian or Alaska Native; Bleed. Disorder, Bleeding disorder; Dis. cancer, Disseminated cancer; Int. Radiology, Interventional Radiology; LOS, Length of hospital stay; Home discharge (+s), Home discharge with services; Mid. Eastern/N. African, Middle Eastern or North African; Nat. Hawaiian/Pacific Isl., Native Hawaiian or Other Pacific Islander.

Adjusted odds ratios (aOR), 95% confidence intervals, and p-values reported; p < 0.05 considered significant.

### Role of the funding source

This research received no external funding. The authors had full independence in the design of the study, collection, analysis, and interpretation of data, writing of the report, and in the decision to submit the paper for publication.

## Results

### Preoperative, intraoperative, and postoperative characteristics

**Fig. 2:**
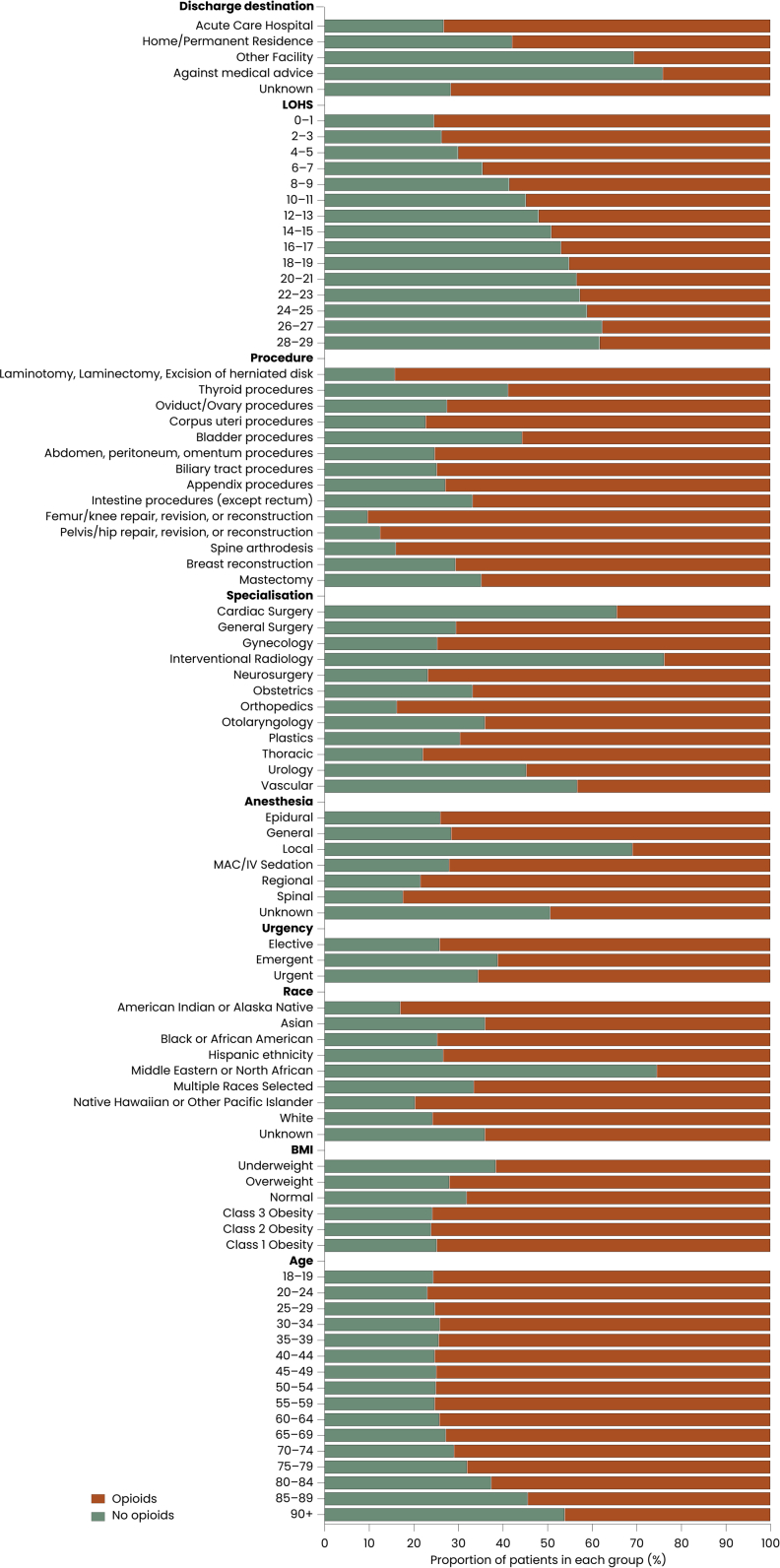
**Distribution of demographic, clinical, procedural, and discharge Characteristics among patients with and without opioid prescription at discharge.** Stacked horizontal bar charts display the proportion of patients discharged with and without an opioid prescription across key demographic and clinical domains, including race, age, surgical specialty, procedure type, discharge destination, and length of hospital stay. Each bar represents the percentage of patients within that category who received an opioid prescription (orange color) compared with those who did not (green color).

Among 945,505 patients, 683,828 (72.3%) were discharged with an opioid prescription. Opioid recipients were slightly younger (mean age 56 vs 58 years, p < 0.0001), had marginally higher Body Mass Index (BMI 31 vs 30 kg/m^2^, p < 0.0001), and differed in race distribution (p < 0.0001) and selected comorbidities (p < 0.0001, for most comparisons; Table 1, Fig. 2). Functional status (p < 0.0001), home support (p = 0.05), and laboratory values (p < 0.0001 for all reported measures) were broadly similar across groups. Although differences reached statistical significance they were small in absolute magnitude.

Intraoperative characteristics varied only modestly (Table 1). Patients receiving opioids had slightly longer operative times [118 (94) vs 114 (99) minutes, p < 0.0001] and were more likely to undergo elective [85% (n = 578,078) vs 77% (n = 201,407), p < 0.001], outpatient surgery [56% (n = 382,503) vs 45% (n = 116,879), p < 0.0001], and orthopedic operations [28% (n = 190,906) vs 14% (n = 36,905), p < 0.0001]. Those not prescribed opioids were more commonly treated in urology [11% (n = 28,649) vs 5.1% (n = 34,657), p < 0.0001], or vascular [5.9% (n = 15,369) vs 1.7% (n = 11,738), p < 0.0001] surgery. Absolute differences across intraoperative variables were generally small despite consistent statistical significance.

Postoperative outcomes showed consistent but small absolute differences (Supplementary Table S2). Patients not discharged with opioids had a longer mean hospital length of stay [3.3 (5.1) vs 2.0 (3.4) days, p < 0.0001]. Rates of 30-day readmission [5.6% (n = 14,695) vs 4.4% (n = 30,132), p < 0.0001] were higher among patients not prescribed opioids. Major complications occurred more frequently in the no-opioid group [11.0% (n = 28,825) vs 7.7% (n = 52,717), p < 0.0001]. Discharge destination differed between groups (p < 0.0001), with patients receiving opioids more frequently discharged home [(n = 655,149, 96% vs n = 239,204, 91%) and patients not receiving opioids more often discharged to another facility [7.7% (n = 20,139) vs 4.1% (n = 27,696)]. Functional decline at discharge was more common among patients not prescribed opioids [5.2% (n = 13,636) vs 4.0% (n = 27,190), p < 0.0001]. Although statistically robust, these outcome differences were modest in absolute terms.

### Procedure specific opioid prescribing patterns

Opioid prescribing at discharge varied substantially across major surgical procedure groups (Fig. 2, Supplementary Table S1). The highest rates occurred in orthopedic and spine procedures, with femur and knee repair or reconstruction having the highest opioid prescription (n = 64,602, 90.3%). Pelvis and hip repair or reconstruction showed similarly high rates (n = 42,101, 87.5%). The lowest prescribing rate among these high-volume procedures was observed following bladder surgery, with 55.7% (n = 31,237) discharged with opioids.

### Postoperative opioid prescription characteristics

Among patients discharged with opioid prescriptions, there was substantial variability in daily dose, cumulative exposure, dosing interval, and medication type (Table 2). Across 471,389 (68.9%) prescriptions with available data, the mean total daily dose was 44.8 MME (SD 122.1; Median: 30; IQR 30–45). For 392,582 (57.4%) prescriptions, the mean cumulative dose over the full prescription period was 197.3 MME (SD 785.5; Median: 105; IQR 75–200). This value represents the average across all surgical specialties and procedures and is disproportionately influenced by high-intensity procedural groups. Most lower-acuity procedures were associated with substantially lower daily MME. Patients received an average of 21 total opioid doses (SD 20.4; Median: 15; IQR 10–28; n = 415,431, 60.8%), and the maximum days supplied averaged 4.2 days (SD 3.3; Median: 3.3; IQR 2.0–5.0; n = 399,093, 58.4%).

Pill formulations predominated (n = 505,546, 99%). Prescription renewals were rare (n = 28,385, 4.2%). Among 478,920 (70%) patients with intensity data, short/low-intensity prescriptions were most common (n = 287,672, 42.1%), followed by high-intensity (n = 119,617, 17.5%) and intermediate-intensity regimens (n = 71,631, 10.5%). In terms of medication type, oxycodone was the predominant agent (n = 427,945, 62.6%).

### Associations between opioid prescription on discharge, hospital stay length, and surgical specialty

**Fig. 3:**
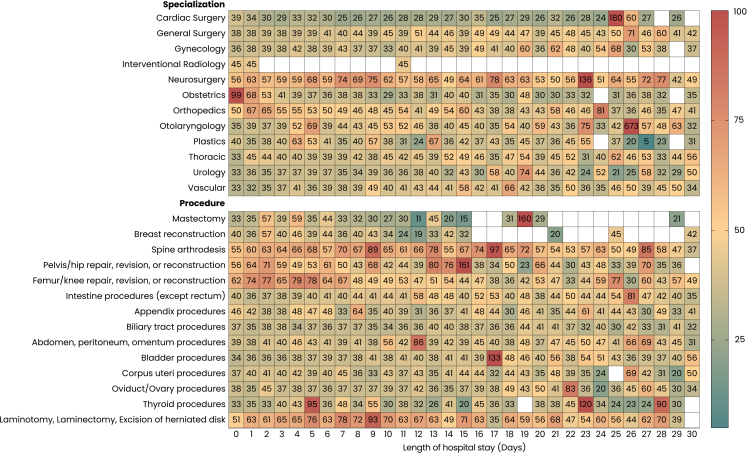
**Mean daily opioid exposure by surgical specialization or procedure and length of stay.** Heatmap of mean daily opioid dosing (mean daily MME) by specialization and procedure. The ten most common procedures from each group (Opioids and No opioids) are included, summing to total of 14 procedures. Each value represents the mean value for a given LOS, with color intensity corresponding to higher opioid prescription (green = low, yellow = moderate, red = high). Neurosurgery and Orthopedics demonstrate the mean daily opioid requirements. Spine arthrodesis, procedures on herniated disks, and repair of the pelvis/hip or femur/knee displayed the highest mean daily MME values.

Postoperative opioid prescription varied across surgical specialties and procedures and increased with longer LOS (Fig. 3, Supplementary Figures S1–S3). Given this heterogeneity, prescribing patterns were examined at the specialty level to avoid overgeneralisation. Neurosurgery and Orthopedics demonstrated the highest opioid exposure, showing high cumulative and mean daily MME at prolonged LOS, with several strata exceeding the upper range of the scale (>350–450 total MME and >50–60 MME/day; Supplementary Figure S2). Lower-acuity specialties, including Gynecology, Urology, Vascular, and Cardiac Surgery, maintained comparatively low and stable opioid requirements across all LOS categories. Patterns were consistent across both cumulative and daily metrics, highlighting distinct specialty-level opioid prescribing profiles (Supplementary Figure S3).

### Multivariable analysis

In adjusted analyses, several variables demonstrated dose–response gradients across intensity levels (Table 3). Increasing age was associated with higher odds of high-intensity opioid prescribing (OR 1.03 per year, 95% CI 1.01–1.04; p < 0.0001) and lower odds of intermediate- (OR 0.93, 95% CI 0.92–0.94; p < 0.0001) and low-intensity opioid prescribing (OR 0.89, 95% CI 0.88–0.89; p < 0.0001).

Compared with White patients, Black patients had lower odds of high intensity (OR 0.91, 95% CI 0.88–0.93; p < 0.0001) but higher odds of low intensity prescribing (OR 1.13, 95% CI 1.11–1.15; p < 0.0001), while Hispanic/Latino patients had low odds across all levels (high intensity OR 0.53, 95% CI 0.43–0.65; p < 0.0001). Markers of frailty and dependency were associated with lower prescribing intensity. Dementia was associated with reduced odds of high (OR 0.66, 95% CI 0.61–0.72; p < 0.0001) and intermediate-intensity prescribing (OR 0.84, 95% CI 0.76–0.93; p = 0.0005). Hypertension was associated with higher odds of high- (OR 1.06, 95% CI 1.04–1.08; p < 0.0001) and low-intensity prescribing (OR 1.05, 95% CI 1.03–1.06; p < 0.0001).

Functional dependence was strongly associated with prescribing patterns. Partially dependent (high intensity OR 0.76, 95% CI 0.71–0.81; p < 0.0001), and totally dependent patients had reduced odds across all intensity categories (high intensity OR 0.59, 95% CI 0.49–0.72; p < 0.0001).

Surgical specialty was among the strongest predictors of prescribing intensity. Compared with general surgery, neurosurgery (OR 5.66, 95% CI 5.50–5.83; p < 0.0001) and orthopedic surgery (OR 6.79, 95% CI 6.64–6.93; p < 0.0001) demonstrated the highest odds of high-intensity opioid prescribing.

Anesthetic technique was independently associated with prescribing intensity. Local anesthesia was associated with increased odds of no opioid prescribing (OR 5.72, 95% CI 4.74–6.91; p < 0.0001), while spinal anesthesia was associated with increased odds of high-intensity prescribing (OR 2.27, 95% CI 2.21–2.33; p < 0.0001).

Major postoperative complications were associated with higher odds of opioid prescribing across intensity categories (high intensity OR 1.23, 95% CI 1.20–1.27; p < 0.0001), and longer postoperative length of stay was associated with increased odds of high-intensity prescribing (OR 1.03 per unit increase, 95% CI 1.02–1.04; p < 0.0001).

### Assessment of missing data and cohort selection

Missing data for preoperative and intraoperative covariates were minimal across most variables. Comparison of the baseline characteristics and postoperative outcomes between patients included in our analytic cohort and those excluded due to missing opioid data or prolonged hospitalization (>30 days) demonstrated systematic differences across multiple demographic, clinical, and outcome variables, with excluded patients being older, more comorbid, and experiencing substantially higher rates of postoperative complications, prolonged mechanical ventilation, and in-hospital mortality (Supplementary Table S3).

## Discussion

The present study examines an important yet incompletely understood aspect of postoperative care: how post-discharge opioid prescribing varies across US surgical practice. Although national initiatives have reduced inpatient opioid exposure, post-discharge prescribing remains comparatively less standardized and continues to show substantial heterogeneity across procedures and specialties.^29^^,^^30^ The transition to home represents a vulnerable period marked by potential misuse, adverse events, and unplanned utilization, underscoring the need for clearer, evidence-based prescribing expectations.^31^ Using a national cohort of 945,505 patients, we found that 72.3% were discharged with an opioid prescription, with substantial variation across procedures and specialties. While multiple patient- and surgery-level factors were statistically associated with prescribing, the dominant sources of variation were structural and practice-related rather than granular indicators of perioperative severity. It is important to note that given the very large cohort size, many associations reached statistical significance despite small absolute differences, underscoring the importance of interpreting these findings in terms of effect size and clinical relevance rather than p-values alone.

Despite heightened attention to opioid stewardship, substantial variation in post-discharge opioid prescribing persists. Existing guidance provides general benchmarks for postoperative prescribing rather than procedure-specific mandates. For example, ACS recommendations commonly suggest 0–20 tablets of 5 mg oxycodone equivalents for many common operations,^32^ and CDC guidance emphasizes use of the lowest effective dose, short prescribing durations, and caution near 50 MME/day.^33^^,^^34^ In our cohort, mean daily dosing (44.8 MME) and cumulative exposure (197 MME) frequently approached or exceeded these thresholds. Refills were uncommon (4.2%), suggesting that many patients received more opioid than necessary, consistent with prior work demonstrating substantial unused postoperative opioid supply.^35^^,^^36^ Together, these findings highlight the continued reliance on precautionary opioid prescribing at discharge and underscore the challenge of translating broad stewardship guidance into consistent, procedure-specific prescribing practices. Such patterns may contribute to excess opioids in the home environment, even in the absence of clear evidence of inadequate pain control.

Prescribing varied widely across specialties, consistent with procedural pain expectations and established service-specific norms.^32^^,^^37^ Orthopedic and spine procedures demonstrated the highest opioid prescription, reflecting recognized postoperative pain intensity and long-standing reliance on opioids.^38^, ^39^, ^40^ Lower prescribing rates were seen in bladder, urologic, otolaryngologic, and cardiac procedures. Even within abdominal and gynecologic surgery, substantial heterogeneity persisted, suggesting that local practice norms, shared order sets, and institutional culture play a significant role.^41^

Multivariable analysis reinforced this interpretation. Specialty and anesthetic technique were among the strongest predictors of high-intensity prescribing, whereas traditional markers of acuity, such as emergent case status, transfusion, or major complications, were only weakly associated and often near neutral. Notably, neuraxial anesthesia was strongly associated with high-intensity prescribing.^42^, ^43^, ^44^ Its use in major orthopedic and spine procedures, combined with anticipated rebound pain as blocks wear off,^45^ may contribute to this association Accordingly, neuraxial anesthesia in this context likely functions as a marker of procedural expectations and service-level prescribing culture rather than as an isolated analgesic determinant. Overall, these findings suggest that postoperative opioid intensity is shaped more by specialty-specific patterns than by granular differences in perioperative severity.

Patient-level demographic and clinical characteristics showed statistically significant but generally small associations with prescribing. Heart failure, kidney disease, ventilator dependence, sepsis, or transfusion demonstrated limited impact on prescribing intensity after accounting for specialty and anesthesia.

Although previous research has described undertreatment of pain among Black and Hispanic patients,^46^^,^^47^ our findings did not demonstrate uniform reductions in opioid exposure. Both groups had lower odds of high-intensity prescribing, but higher odds of any opioid prescribing and higher odds of low-intensity prescribing. Patients identifying as Middle Eastern or North African showed notably lower odds across exposure levels, suggesting potentially distinct prescribing patterns. These differences may reflect procedural mix, implicit bias, cultural preferences, or unmeasured socioeconomic factors,^48^^,^^49^ limitations that cannot be fully addressed within the NSQIP.

The overall racial and ethnic distribution of our cohort broadly reflects national patterns but deviates in important ways. White patients comprise 60% of the study population, which is slightly lower than their proportion in the contemporary US adult population, while Black or African American patients account for 9.9%, also somewhat lower than national estimates (∼12–13%). Asian patients (3.8%) and American Indian or Alaska Native patients (0.7%) are also underrepresented relative to US census data. Notably, nearly one-quarter of patients (24%) have unknown race, which exceeds national nonresponse rates and likely reflects health system-level differences in data collection rather than true population structure. Hispanic ethnicity is reported in 11% of the cohort, which is substantially lower than population estimates (∼18–19%), suggesting systematic undercapture or misclassification of ethnicity. This limitation is particularly relevant given known disparities in perioperative care and pain management among Hispanic populations and may bias estimates toward the null or obscure meaningful differences.

These discrepancies likely reflect the nature of the data source rather than sampling error alone. Surgical registries and hospital-based datasets disproportionately capture patients with access to operative care, stable insurance coverage, and engagement with large health systems, and may underrepresent younger, uninsured, rural, or socioeconomically marginalized populations. As a result, while the cohort is large and geographically diverse, it should be interpreted as representative of patients undergoing surgery within US health systems, rather than the US population at large.

Importantly, because race and ethnicity are sociocultural constructs, observed differences in opioid exposure across groups should be interpreted in the context of structural and systemic drivers rather than intrinsic patient characteristics. Residual confounding by unmeasured factors, such as socioeconomic status, insurance type, geographic region, hospital characteristics, and clinician-level prescribing practices, likely contributes to observed variations. The small effect sizes (Cramér's V ≤ 0.08) further indicate that while statistically significant, race and ethnicity explain only a limited proportion of variance in opioid prescribing patterns. Conclusions regarding equity must remain cautious and should not be interpreted as definitive evidence of disparities without further study.

As in prior studies, patients discharged with opioids had shorter hospital stays (2.0 vs 3.3 days) and fewer major complications than those discharged without them. Although this appears inconsistent with known opioid-related risks,^33^^,^^49^^,^^50^ these patterns almost certainly reflect patient selection rather than protective effects. Specifically, opioid prescribing at discharge in this context should be understood primarily as a marker of clinical stability and recovery trajectory rather than as a modifiable exposure with causal benefit. Patients with uncomplicated recoveries are more likely to be discharged earlier and considered appropriate for oral opioid therapy, whereas those with complications, sepsis, renal dysfunction, or delirium typically require longer hospitalization, parenteral analgesia, and therefore are less likely to receive opioids at discharge.

Multivariable analyses support this interpretation. Major complications were associated with only modest increases in the odds of receiving any opioid intensity, while markers of severe or destabilizing events (delirium and postoperative oxygen use) were generally linked to lower or minimally changed odds of high-intensity prescribing. These small effect sizes, in the context of a large cohort, indicate that postoperative severity explains little of the variation in opioid intensity.

Taken together, these findings reinforce that observed associations between opioid prescribing and favorable early outcomes reflect reverse causality, patients who recover smoothly leave the hospital sooner and receive oral opioids, rather than opioid-related benefit.

This pattern is particularly evident in delirium outcomes. Patients not prescribed opioids had much higher rates of postoperative delirium screening and diagnosis, reflecting ascertainment and selection bias rather than reduced risk. Patients with cognitive concerns are monitored more closely and are less likely to receive opioids.^51^^,^^52^ Thus, the inverse association between opioid prescribing and adverse outcomes should not be interpreted as evidence of benefit but rather as a marker of clinical stability and recovery trajectory.

Although US prescribing has declined from earlier rates exceeding 90%, the 72% discharge opioid rate observed here remains far higher than international norms. The iPOP study reported opioid use in 91.6% of US patients compared with 5.1% abroad in 2020, with a >40-fold difference in MME supplied.^53^ Many countries with comparable surgical complexity emphasize multimodal analgesia and reserve opioids for breakthrough pain.^32^^,^^41^^,^^53^^,^^54^ Despite progress, US practice continues to rely heavily on precautionary opioid prescribing.

The notable heterogeneity across specialties and procedures highlights opportunities to improve postoperative pain management. Aligning prescribing with procedure-specific evidence and incorporating recommended quantities into order sets and discharge pathways may reduce excess opioid supply while maintaining adequate analgesia.^55^^,^^56^ Benchmarking tools that compare prescribing against guideline targets may further promote consistency.^32^

Given the relatively weak influence of disease severity on prescribing intensity, interventions targeting specialty-level culture, shared order sets, cross-specialty consensus, and transparent feedback, may be particularly impactful. Reducing unnecessary exposure will also require greater reliance on multimodal analgesia, careful reassessment before issuing large prescriptions, and normalization of refill-based rather than precautionary prescribing.

This study has several strengths, including its large national sample, detailed discharge prescribing data, and procedure-level stratification, which allow meaningful comparisons across specialties. The inclusion of postoperative complications and discharge disposition measures also supports assessment of early clinical outcomes. However, several limitations must be acknowledged.

As a retrospective registry analysis, causal inference cannot be established. The absence of hospital identifiers in NSQIP prevents evaluation of site-level variation or clustering, limiting assessment of institutional prescribing culture or stewardship efforts. The dataset also lacks a dedicated measure of preoperative opioid use, preventing differentiation between opioid-naïve patients and those on chronic therapy and potentially confounding associations between prescribing and outcomes. Baseline opioid tolerance may meaningfully influence both prescribing decisions and apparent prescribing intensity. Patients with preoperative opioid exposure often require higher daily MME, longer treatment durations, and more frequent prescription renewals to achieve comparable analgesia, independent of surgical complexity or immediate postoperative course. This limitation is particularly relevant when interpreting specialty-level variation. Orthopedic and spine surgery populations have a higher prevalence of chronic pain and preoperative opioid use, which may partially account for the greater prescribing intensity observed in these cohorts. Although we adjusted for operative complexity, length of stay, complications, and discharge destination as proxies for clinical trajectory, residual confounding related to unmeasured baseline opioid exposure likely persists. Because the NSQIP captures only 30-day postoperative data, longer-term opioid-related sequelae, such as persistent postoperative use or dose escalation, could not be assessed.

Missing opioid data were not missing completely at random, and likely reflect differential loss of patients with severe or prolonged postoperative courses who did not reach a typical discharge state or lacked complete documentation. We restricted our primary analyses to patients with complete post-discharge prescribing data and interpret all estimates as applying to this clinically-relevant subset of surgical patients who survived to discharge with documented analgesic plans.

Delirium ascertainment may be biased, as screening practices vary across institutions, complicating interpretation of delirium-related findings.^57^ The NSQIP does not capture outpatient or emergency department visits, which may underestimate short-term opioid-related morbidity. These limitations highlight the need for prospective, multi-center studies with more complete measures of opioid exposure, patient-reported recovery, and extended follow-up to improve risk stratification and support safer, guideline-concordant prescribing.

## Contributors

ACP conceptualized the study, contributed to study design, oversaw data acquisition and analysis, interpreted the data, and drafted the manuscript. DYM and TS contributed to data curation, statistical analysis, and interpretation of results. TN and JB assisted with data management, literature review, and validation of analyses. IG contributed to clinical interpretation of findings and critical revision of the manuscript for important intellectual content. DPO contributed to study design, provided senior methodological and clinical oversight, and critically revised the manuscript. GH supervised the project, contributed to study design and data interpretation, critically revised the manuscript for intellectual content and takes final responsibility for the decision to submit for publication.

## Data sharing statement

The data used in this study were obtained from the American College of Surgeons National Surgical Quality Improvement Program (ACS-NSQIP). These data are not publicly available due to data use agreements and institutional restrictions designed to protect patient confidentiality. Access to ACS-NSQIP Participant Use Data Files (PUFs) may be granted to investigators affiliated with participating institutions who obtain approval from the American College of Surgeons and execute the required data use agreements. Information on data access and application procedures is available from the American College of Surgeons at https://www.facs.org/quality-programs/data-and-registries/acs-nsqip.

## Declaration of interests

The authors declare no competing interests and have no financial interest in any of the products, devices, or drugs mentioned in this manuscript.
