# Supplementary material for: Post-discharge opioid prescribing after surgery in the United States: a population-based analysis of specialty variation and prescribing intensity
Source: Lancet Reg Health Am. 2026 Mar 13;57:101456. doi: 10.1016/j.lana.2026.101456 (PMC12999286; doi:10.1016/j.lana.2026.101456)
Supplement: Supplementary Material [file mmc1.pdf]

Supplementary Material

Contents

1. **Figures**

   1.1. Supplementary Figure 1

   1.2. Supplementary Figure 2

   1.3. Supplementary Figure 3

2. **Tables**

   2.2. Supplementary Table 1

   2.3. Supplementary Table 2

   2.4. Supplementary Table 3

1. Figures

1.1. Supplementary Figure 1.

Mean daily morphine milligram equivalents (MME) across a wide range of surgical procedure groups stratified by hospital length of stay (LOS). Each cell represents the average MME prescribed at discharge for patients undergoing procedures within a given CPT code range and LOS duration from 0–30 days. Warmer colors indicate higher opioid exposure, while cooler colors indicate lower exposure. This heatmap illustrates substantial inter- and intra-procedural variation in discharge opioid prescribing, even among procedures with similar LOS profiles.

Mean daily morphine milligram equivalents (MME) across a wide range of surgical procedure groups stratified by hospital length of stay (LOS). Each cell represents the average MME prescribed at discharge for patients undergoing procedures within a given CPT code range and LOS duration from 0–30 days. Warmer colors indicate higher opioid exposure, while cooler colors indicate lower exposure. This heatmap illustrates substantial inter- and intra-procedural variation in discharge opioid prescribing, even among procedures with similar LOS profiles.

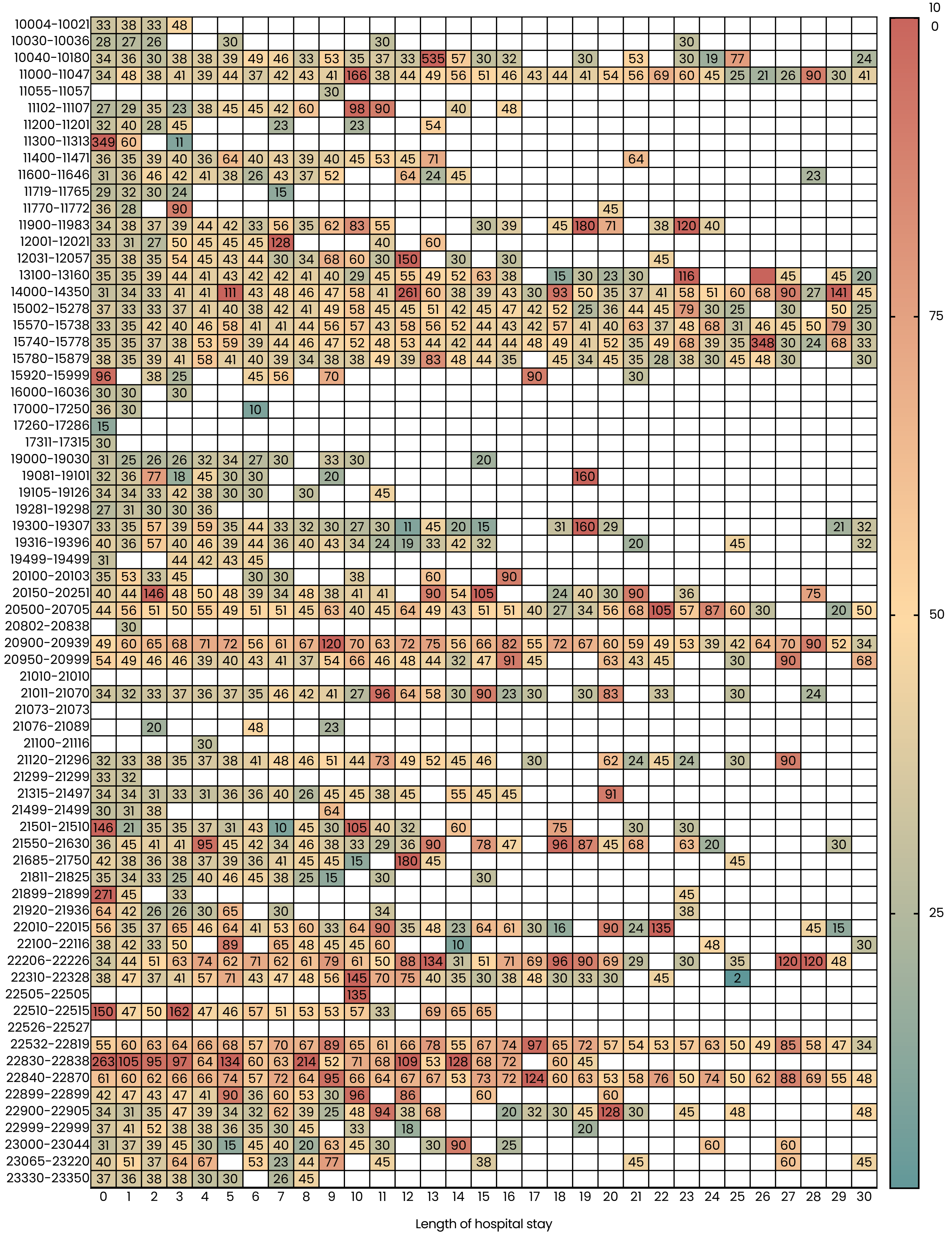
10004-10021 33 38 33 48
10030-10036 28 27 26 30 30 30
10040-10180 34 36 30 38 38 39 49 46 33 53 35 37 33 535 57 30 32 30 53 30 19 77 24
11000-11047 34 48 38 41 39 44 37 42 43 41 166 38 44 49 56 51 46 43 44 41 54 56 69 60 45 25 21 26 90 30 41
11055-11057 30
11102-11107 27 29 35 23 38 45 45 42 60 98 90 40 48
11200-11201 32 40 28 45 23 23 54
11300-11313 349 60 11
11400-11471 36 35 39 40 36 64 40 43 39 40 45 53 45 71 64
11600-11646 31 36 46 42 41 38 26 43 37 52 64 24 45 23
11719-11765 29 32 30 24 15
11770-11772 36 28 90 45
11900-11983 34 38 37 39 44 42 33 56 35 62 83 55 30 39 45 180 71 38 120 40
12001-12021 33 31 27 50 45 45 45 128 40 60
12031-12057 35 38 35 54 45 43 44 30 34 68 60 30 150 30 30 45
13100-13160 35 35 39 44 41 43 42 42 41 40 29 45 55 49 52 63 38 15 30 23 30 116 45 45 20
14000-14350 31 34 33 41 41 111 43 48 46 47 58 41 261 60 38 39 43 30 93 50 35 37 41 58 51 60 68 90 27 141 45
15002-15278 37 33 33 37 41 40 38 42 41 49 58 45 45 51 42 45 47 42 52 25 36 44 45 79 30 25 30 50 25
15570-15738 33 35 42 40 46 58 41 41 44 56 57 43 58 56 52 44 43 42 57 41 40 63 37 48 68 31 46 45 50 79 30
15740-15778 35 35 37 38 53 59 39 44 46 47 52 48 53 44 42 44 44 48 49 41 52 35 49 68 39 35 348 30 24 68 33
15780-15879 38 35 39 41 58 41 40 39 34 38 38 49 39 83 48 44 35 45 34 45 35 28 38 30 45 48 30 30
15920-15999 96 38 25 45 56 70 90 30
16000-16036 30 30 30
17000-17250 36 30 10
17260-17286 15
17311-17315 30
19000-19030 31 25 26 26 32 34 27 30 33 30 20
19081-19101 32 36 77 18 45 30 30 20 160
19105-19126 34 34 33 42 38 30 30 30 45
19281-19298 27 31 30 30 36
19300-19307 33 35 57 39 59 35 44 33 32 30 27 30 11 45 20 15 31 160 29 21 32
19316-19396 40 36 57 40 46 39 44 36 40 43 34 24 19 33 42 32 20 45 32
19499-19499 31 44 42 43 45
20100-20103 35 53 33 45 30 30 38 60 90
20150-20251 40 44 146 48 50 48 39 34 48 38 41 41 90 54 105 24 40 30 90 36 75
20500-20705 44 56 51 50 55 49 51 51 45 63 40 45 64 49 43 51 51 40 27 34 56 68 105 57 87 60 30 20 50
20802-20838 30
20900-20939 49 60 65 68 71 72 56 61 67 120 70 63 72 75 56 66 82 55 72 67 60 59 49 53 39 42 64 70 90 52 34
20950-20999 54 49 46 46 39 40 43 41 37 54 66 46 48 44 32 47 91 45 63 43 45 30 90 68
21010-21010
21011-21070 34 32 33 37 36 37 35 46 42 41 27 96 64 58 30 90 23 30 30 83 33 30 24
21073-21073
21076-21089 20 48 23
21100-21116 30
21120-21296 32 33 38 35 37 38 41 48 46 51 44 73 49 52 45 46 30 62 24 45 24 30 90
21299-21299 33 32
21315-21497 34 34 31 33 31 36 36 40 26 45 45 38 45 55 45 45 91
21499-21499 30 31 38 64
21501-21510 146 21 35 35 37 31 43 10 45 30 105 40 32 60 75 30 30
21550-21630 36 45 41 41 95 45 42 34 46 38 33 29 36 90 78 47 96 87 45 68 63 20 30
21685-21750 42 38 36 38 37 39 36 41 45 45 15 180 45 45
21811-21825 35 34 33 25 40 46 45 38 25 15 30 30
21899-21899 271 45 33 45
21920-21936 64 42 26 26 30 65 30 34 38
22010-22015 56 35 37 65 46 64 41 53 60 33 64 90 35 48 23 64 61 30 16 90 24 135 45 15
22100-22116 38 42 33 50 89 65 48 45 45 60 10 48 30
22206-22226 34 44 51 63 74 62 71 62 61 79 61 50 88 134 31 51 71 69 96 90 69 29 30 35 120 120 48
22310-22328 38 47 37 41 57 71 43 47 48 56 145 70 75 40 35 30 38 48 30 33 30 45 2
22505-22505 135
22510-22515 150 47 50 162 47 46 57 51 53 53 57 33 69 65 65
22526-22527
22532-22819 55 60 63 64 66 68 57 70 67 89 65 61 66 78 55 67 74 97 65 72 57 54 53 57 63 50 49 85 58 47 34
22830-22838 263 105 95 97 64 134 60 63 214 52 71 68 109 53 128 68 72 60 45
22840-22870 61 60 62 66 66 74 57 72 64 95 66 64 67 67 53 73 72 124 60 63 53 58 76 50 74 50 62 88 69 55 48
22899-22899 42 47 43 47 41 90 36 60 53 30 96 86 60 60
22900-22905 34 31 35 47 39 34 32 62 39 25 48 94 38 68 20 32 30 45 128 30 45 48 48
22999-22999 37 41 52 38 38 36 35 30 45 33 18 20
23000-23044 31 37 39 45 30 15 45 40 20 63 45 30 30 90 25 60 60
23065-23220 40 51 37 64 67 53 23 44 77 45 38 45 60 45
23330-23350 37 36 38 38 30 30 26 45
0 1 2 3 4 5 6 7 8 9 10 11 12 13 14 15 16 17 18 19 20 21 22 23 24 25 26 27 28 29 30
Length of hospital stay
10
0
75
50
25

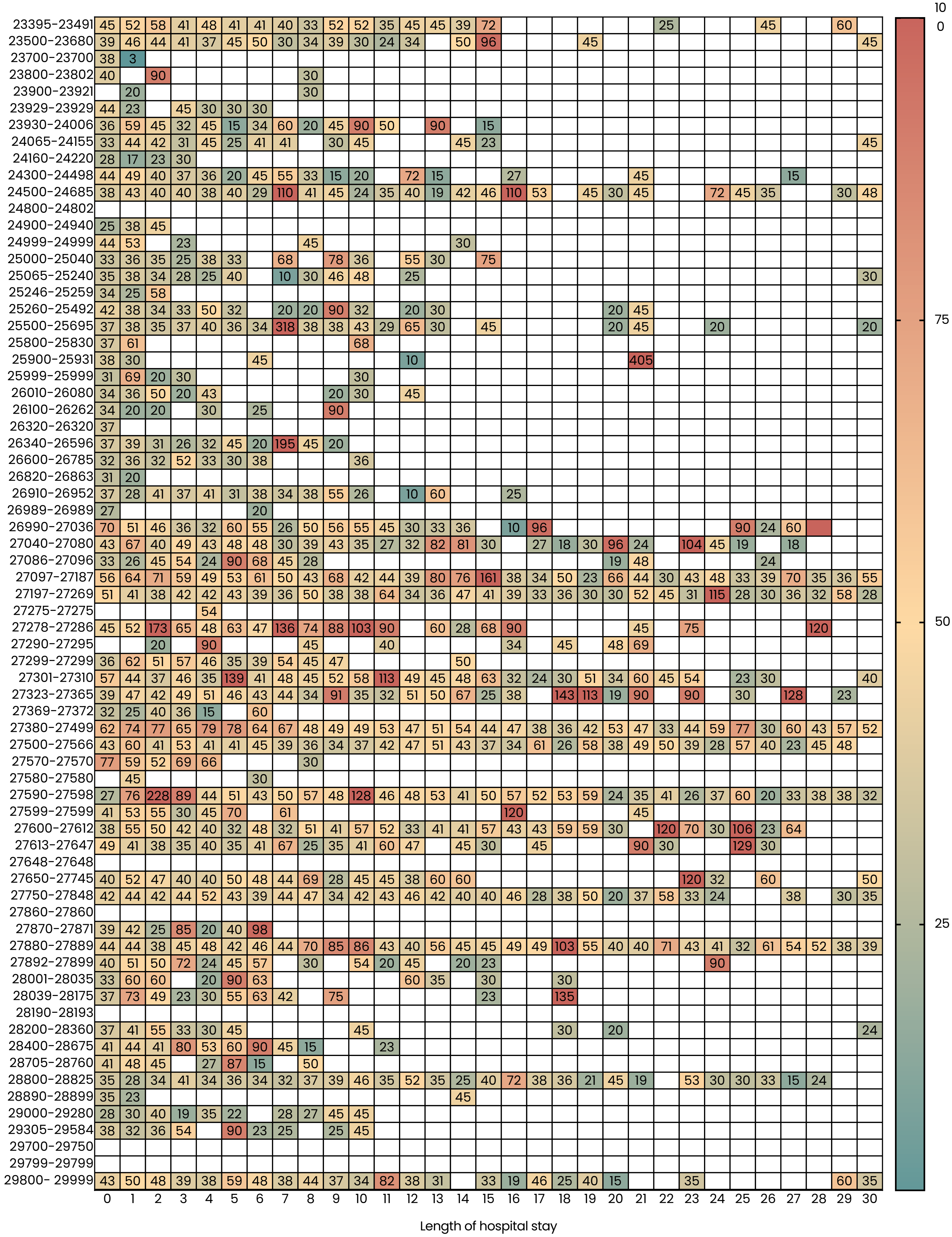
23395-23491
23500-23680
23700-23700
23800-23802
23900-23921
23929-23929
23930-24006
24065-24155
24160-24220
24300-24498
24500-24685
24800-24802
24900-24940
24999-24999
25000-25040
25065-25240
25246-25259
25260-25492
25500-25695
25800-25830
25900-25931
25999-25999
26010-26080
26100-26262
26320-26320
26340-26596
26600-26785
26820-26863
26910-26952
26989-26989
26990-27036
27040-27080
27086-27096
27097-27187
27197-27269
27275-27275
27278-27286
27290-27295
27299-27299
27301-27310
27323-27365
27369-27372
27380-27499
27500-27566
27570-27570
27580-27580
27590-27598
27599-27599
27600-27612
27613-27647
27648-27648
27650-27745
27750-27848
27860-27860
27870-27871
27880-27889
27892-27899
28001-28035
28039-28175
28190-28193
28200-28360
28400-28675
28705-28760
28800-28825
28890-28899
29000-29280
29305-29584
29700-29750
29799-29799
29800- 29999
0 1 2 3 4 5 6 7 8 9 10 11 12 13 14 15 16 17 18 19 20 21 22 23 24 25 26 27 28 29 30
Length of hospital stay
10
0
75
50
25

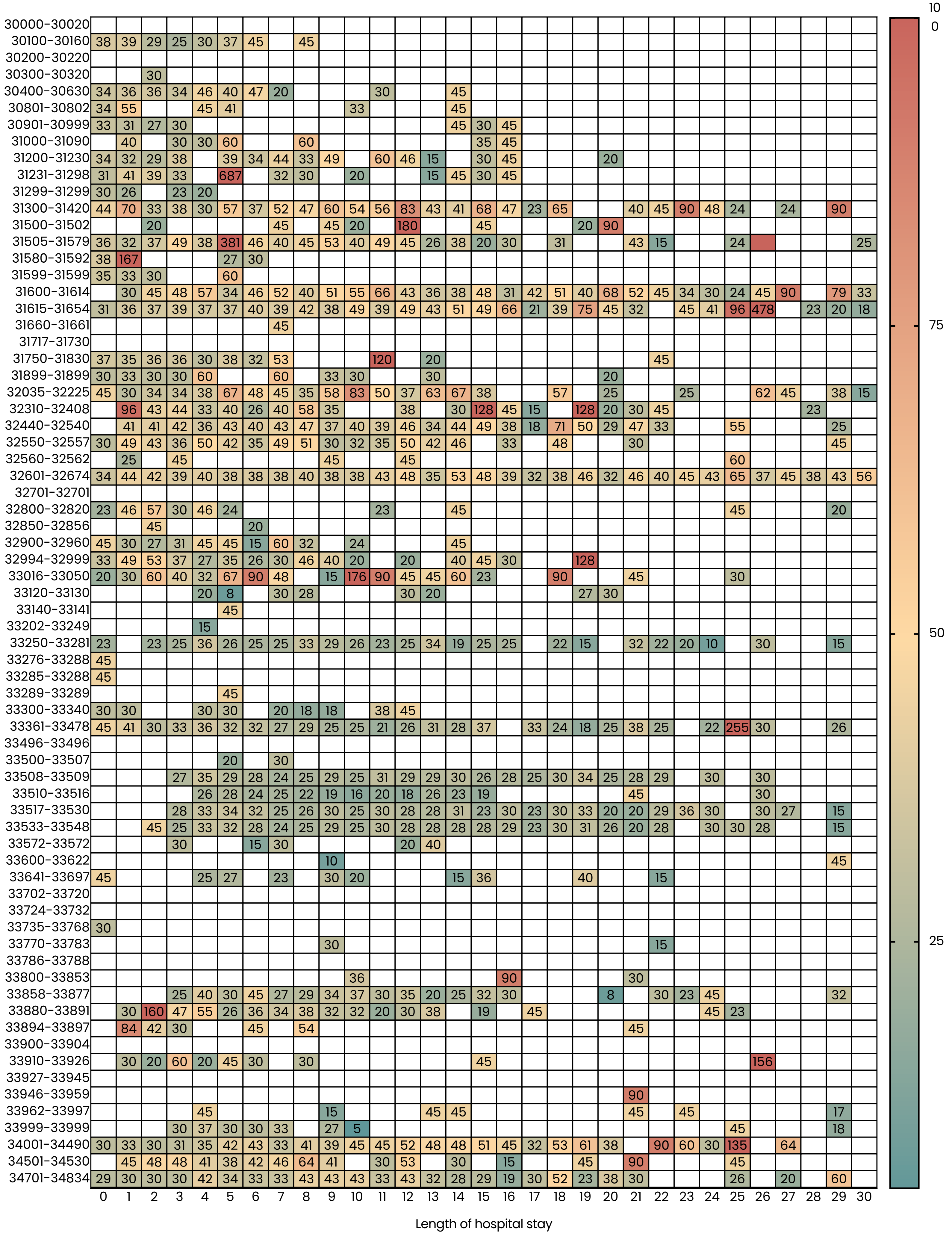
30000-30020
30100-30160
30200-30220
30300-30320
30400-30630
30801-30802
30901-30999
31000-31090
31200-31230
31231-31298
31299-31299
31300-31420
31500-31502
31505-31579
31580-31592
31599-31599
31600-31614
31615-31654
31660-31661
31717-31730
31750-31830
31899-31899
32035-32225
32310-32408
32440-32540
32550-32557
32560-32562
32601-32674
32701-32701
32800-32820
32850-32856
32900-32960
32994-32999
33016-33050
33120-33130
33140-33141
33202-33249
33250-33281
33276-33288
33285-33288
33289-33289
33300-33340
33361-33478
33496-33496
33500-33507
33508-33509
33510-33516
33517-33530
33533-33548
33572-33572
33600-33622
33641-33697
33702-33720
33724-33732
33735-33768
33770-33783
33786-33788
33800-33853
33858-33877
33880-33891
33894-33897
33900-33904
33910-33926
33927-33945
33946-33959
33962-33997
33999-33999
34001-34490
34501-34530
34701-34834
0 1 2 3 4 5 6 7 8 9 10 11 12 13 14 15 16 17 18 19 20 21 22 23 24 25 26 27 28 29 30
Length of hospital stay
10
0
75
50
25

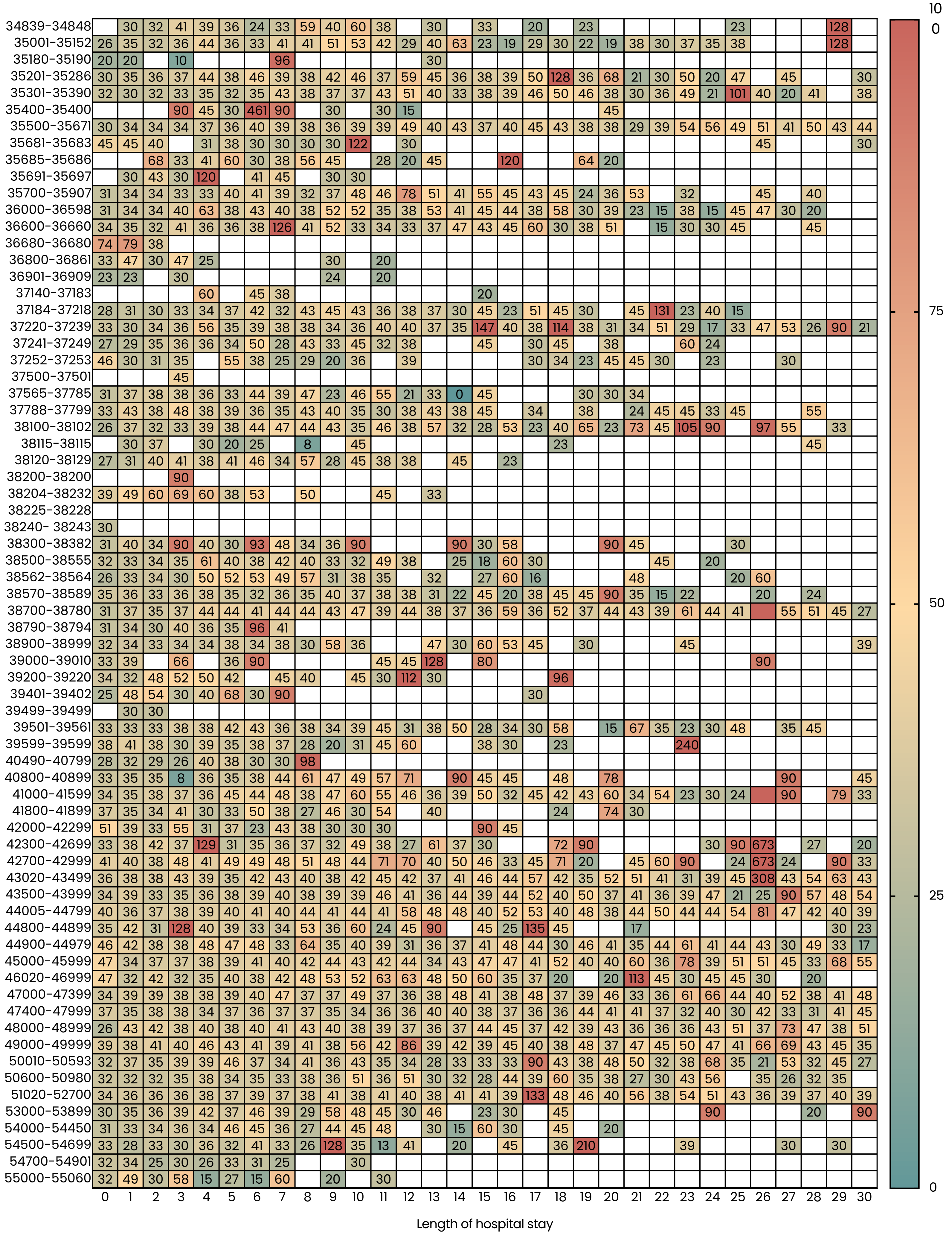
34839-34848
35001-35152
35180-35190
35201-35286
35301-35390
35400-35400
35500-35671
35681-35683
35685-35686
35691-35697
35700-35907
36000-36598
36600-36660
36680-36680
36800-36861
36901-36909
37140-37183
37184-37218
37220-37239
37241-37249
37252-37253
37500-37501
37565-37785
37788-37799
38100-38102
38115-38115
38120-38129
38200-38200
38204-38232
38225-38228
38240- 38243
38300-38382
38500-38555
38562-38564
38570-38589
38700-38780
38790-38794
38900-38999
39000-39010
39200-39220
39401-39402
39499-39499
39501-39561
39599-39599
40490-40799
40800-40899
41000-41599
41800-41899
42000-42299
42300-42699
42700-42999
43020-43499
43500-43999
44005-44799
44800-44899
44900-44979
45000-45999
46020-46999
47000-47399
47400-47999
48000-48999
49000-49999
50010-50593
50600-50980
51020-52700
53000-53899
54000-54450
54500-54699
54700-54901
55000-55060
0 1 2 3 4 5 6 7 8 9 10 11 12 13 14 15 16 17 18 19 20 21 22 23 24 25 26 27 28 29 30
Length of hospital stay
100
75
50
25
0

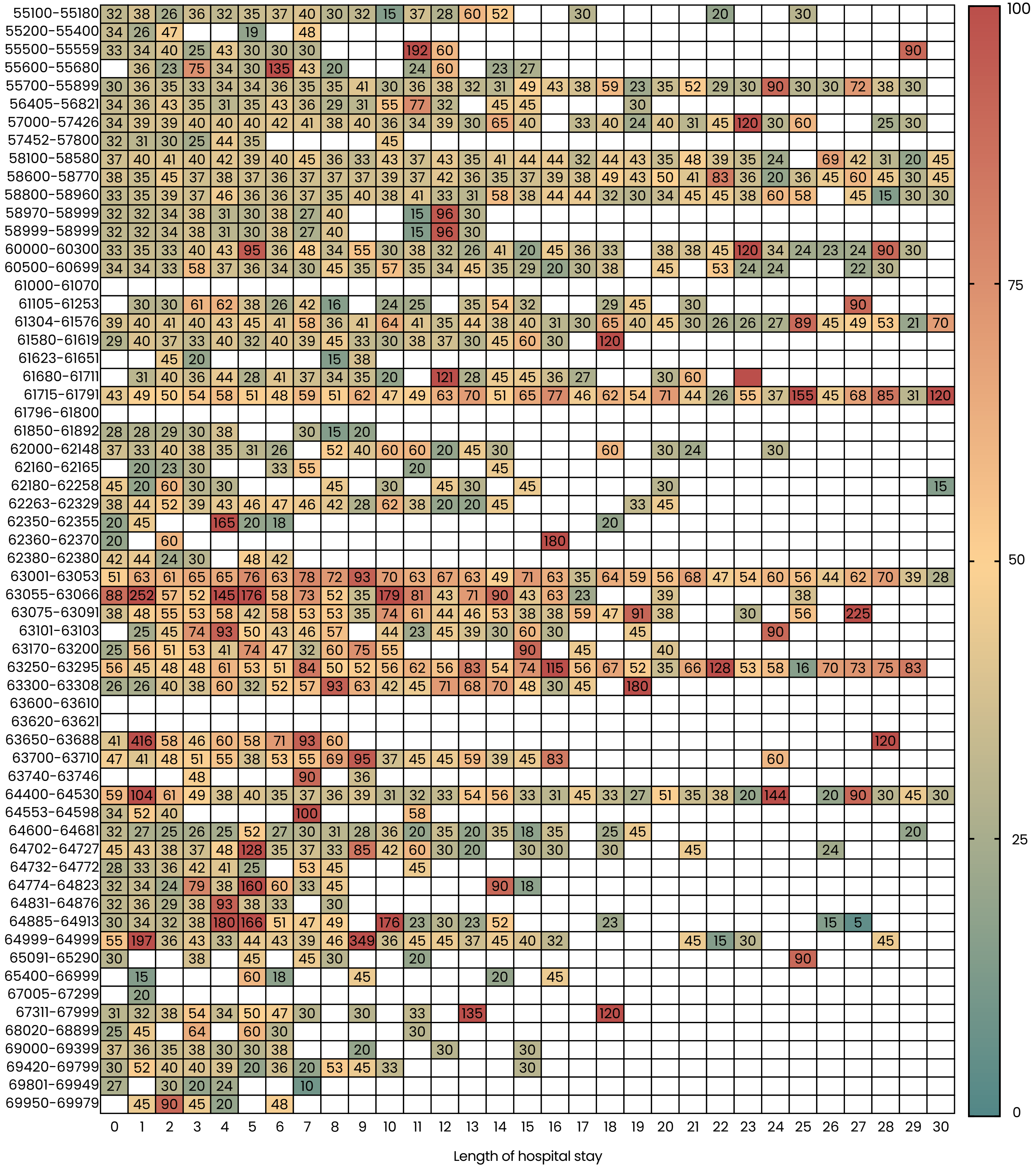
55100-55180
55200-55400
55500-55559
55600-55680
55700-55899
56405-56821
57000-57426
57452-57800
58100-58580
58600-58770
58800-58960
58970-58999
58999-58999
60000-60300
60500-60699
61000-61070
61105-61253
61304-61576
61580-61619
61623-61651
61680-61711
61715-61791
61796-61800
61850-61892
62000-62148
62160-62165
62180-62258
62263-62329
62350-62355
62360-62370
62380-62380
63001-63053
63055-63066
63075-63091
63101-63103
63170-63200
63250-63295
63300-63308
63600-63610
63620-63621
63650-63688
63700-63710
63740-63746
64400-64530
64553-64598
64600-64681
64702-64727
64732-64772
64774-64823
64831-64876
64885-64913
64999-64999
65091-65290
65400-66999
67005-67299
67311-67999
68020-68899
69000-69399
69420-69799
69801-69949
69950-69979
0 1 2 3 4 5 6 7 8 9 10 11 12 13 14 15 16 17 18 19 20 21 22 23 24 25 26 27 28 29 30
Length of hospital stay
100
75
50
25
0

1.2. Supplementary Figure 2.

Heatmap of total morphine milligram equivalents (MME) prescribed at discharge across major surgical specialties stratified by postoperative hospital length of stay (LOS). Each cell represents the cumulative opioid exposure for patients within a given specialty and LOS duration from 0–30 days. M is the mean across the specialty. Warmer colors denote higher total MME. The figure demonstrates substantial heterogeneity in discharge prescribing intensity across specialties, with neurosurgery and orthopedics consistently associated with the highest cumulative exposure across multiple LOS strata, while urology and otolaryngology exhibit comparatively lower prescribing levels.

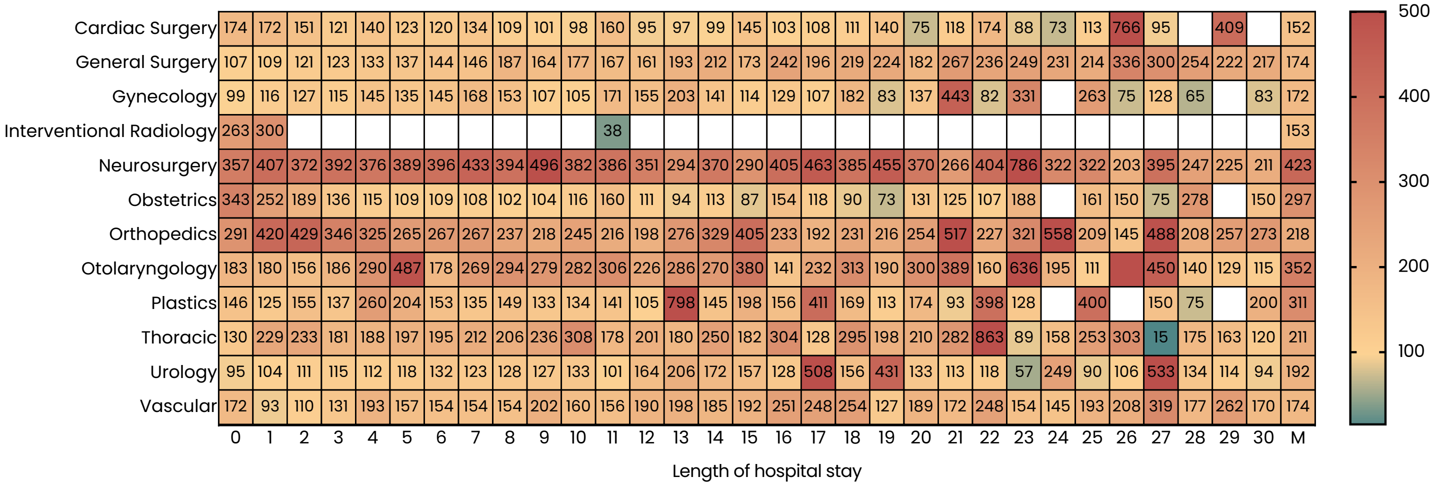

1.3. Supplementary Figure 3.

Heatmap of total morphine milligram equivalents (MME) prescribed at discharge across all major CPT code groupings stratified by postoperative hospital length of stay (LOS). Each cell represents the mean cumulative opioid exposure for patients undergoing procedures within the specified CPT code range and LOS category (0–30 days). M is the mean across the CPT code. Warmer colors reflect higher opioid exposure. This granular procedure-level visualization demonstrates marked heterogeneity in prescribing intensity both across and within surgical procedure categories, revealing wide variation even among operations with similar LOS trajectories.

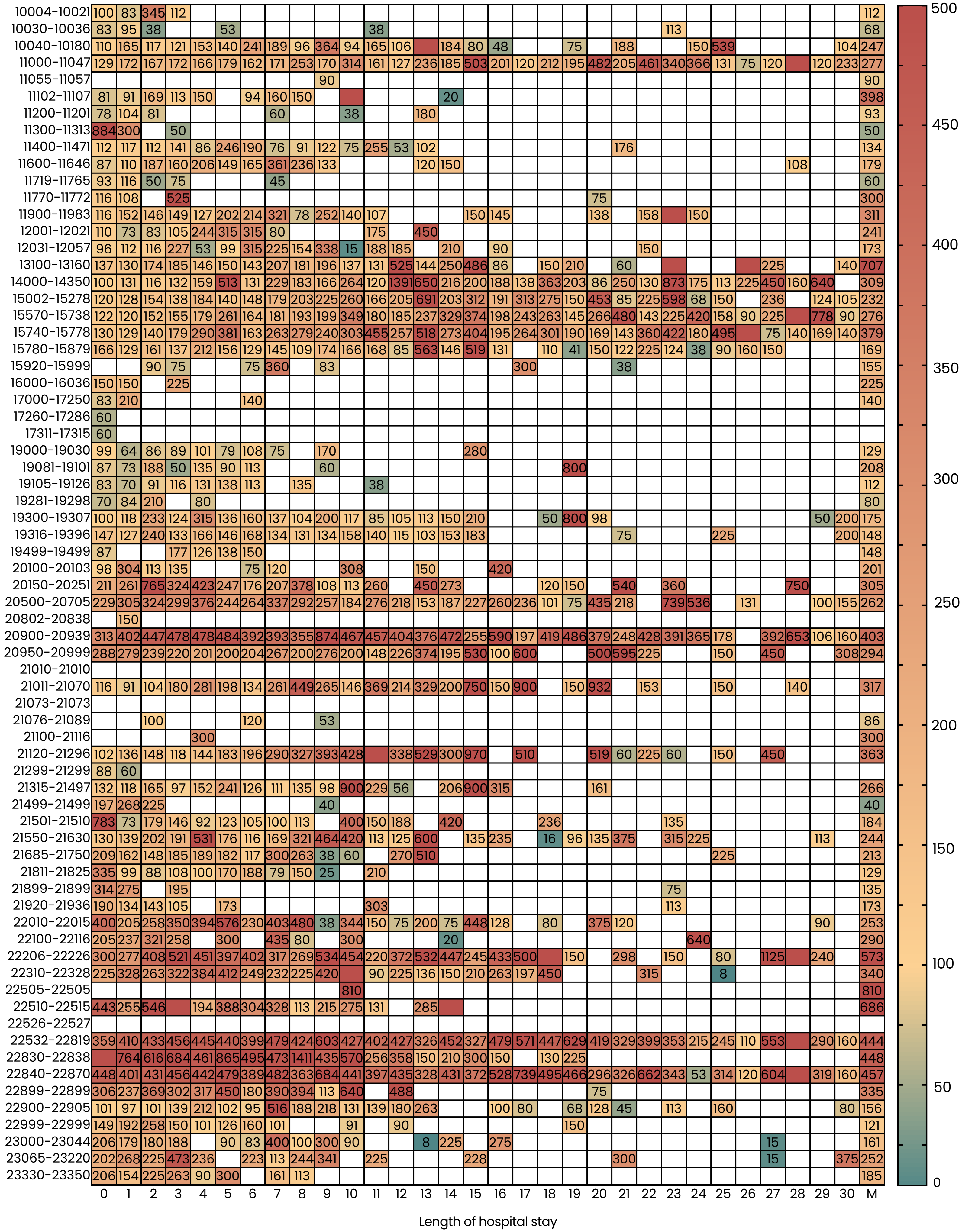

10004-10021
10030-10036
10040-10180
11000-11047
11055-11057
11102-11107
11200-11201
11300-11313
11400-11471
11600-11646
11719-11765
11770-11772
11900-11983
12001-12021
12031-12057
13100-13160
14000-14350
15002-15278
15570-15738
15740-15778
15780-15879
15920-15999
16000-16036
17000-17250
17260-17286
17311-17315
19000-19030
19081-19101
19105-19126
19281-19298
19300-19307
19316-19396
19499-19499
20100-20103
20150-20251
20500-20705
20802-20838
20900-20939
20950-20999
21010-21010
21011-21070
21073-21073
21076-21089
21100-21116
21120-21296
21299-21299
21315-21497
21499-21499
21501-21510
21550-21630
21685-21750
21811-21825
21899-21899
21920-21936
22010-22015
22100-22116
22206-22226
22310-22328
22505-22505
22510-22515
22526-22527
22532-22819
22830-22838
22840-22870
22899-22899
22900-22905
22999-22999
23000-23044
23065-23220
23330-23350
0 1 2 3 4 5 6 7 8 9 10 11 12 13 14 15 16 17 18 19 20 21 22 23 24 25 26 27 28 29 30 M
Length of hospital stay
500
450
400
350
300
250
200
150
100
50
0

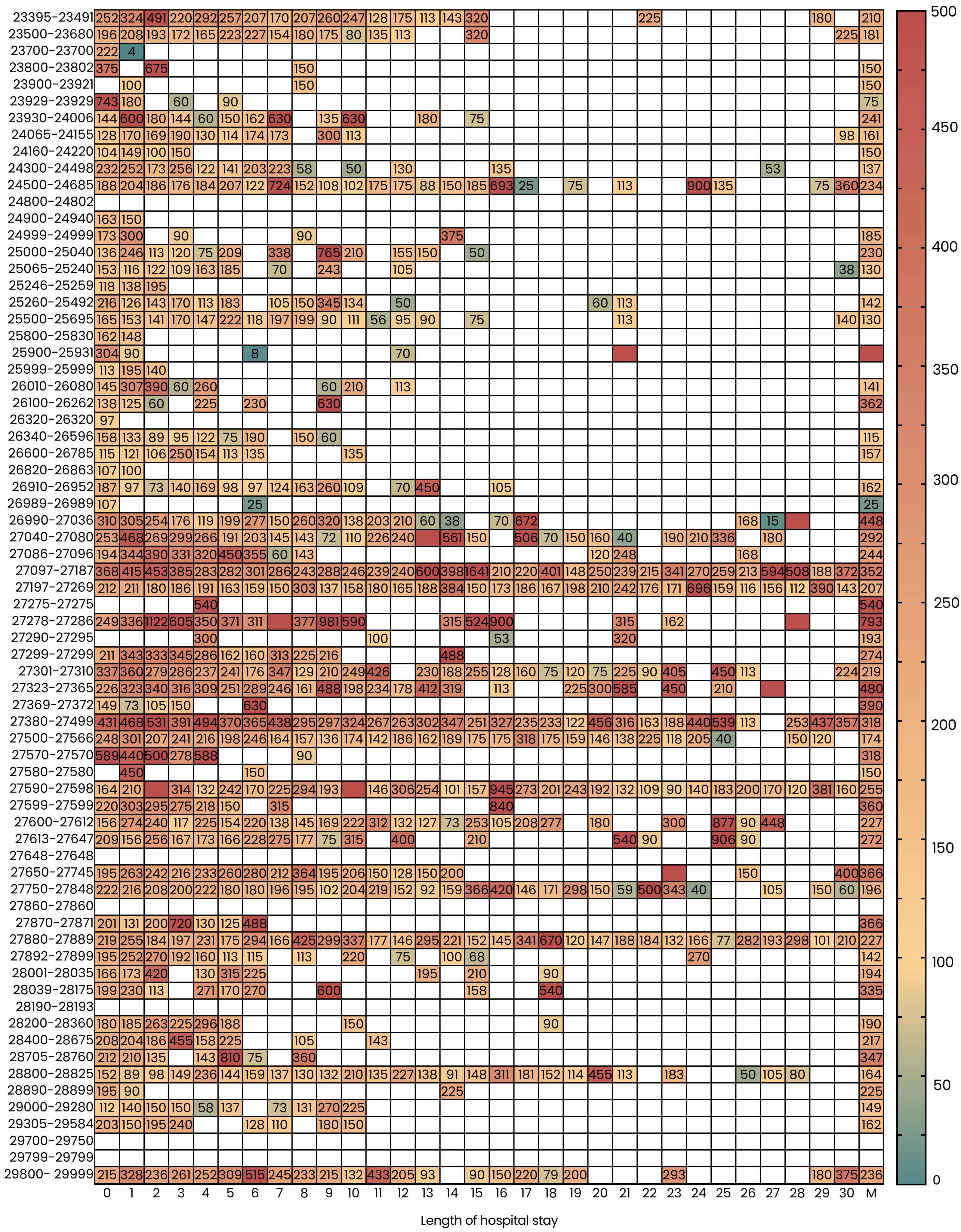
23395-23491 252 324 491 220 292 257 207 170 207 260 247 128 175 113 143 320 225 180 210
23500-23680 196 208 193 172 165 223 227 154 180 175 80 135 113 320 225 181
23700-23700 222 4
23800-23802 375 675 150 150
23900-23921 100 150 150
23929-23929 743 180 60 90 75
23930-24006 144 600 180 144 60 150 162 630 135 630 180 75 241
24065-24155 128 170 169 190 130 114 174 173 300 113 98 161
24160-24220 104 149 100 150 150
24300-24498 232 252 173 256 122 141 203 223 58 50 130 135 53 137
24500-24685 188 204 186 176 184 207 122 724 152 108 102 175 175 88 150 185 693 25 75 113 900 135 75 360 234
24800-24802
24900-24940 163 150
24999-24999 173 300 90 90 375 185
25000-25040 136 246 113 120 75 209 338 765 210 155 150 50 230
25065-25240 153 116 122 109 163 185 70 243 105 38 130
25246-25259 118 138 195
25260-25492 216 126 143 170 113 183 105 150 345 134 50 60 113 142
25500-25695 165 153 141 170 147 222 118 197 199 90 111 56 95 90 75 113 140 130
25800-25830 162 148
25900-25931 304 90 8 70
25999-25999 113 195 140
26010-26080 145 307 390 60 260 60 210 113 141
26100-26262 138 125 60 225 230 630 362
26320-26320 97
26340-26596 158 133 89 95 122 75 190 150 60 115
26600-26785 115 121 106 250 154 113 135 135 157
26820-26863 107 100
26910-26952 187 97 73 140 169 98 97 124 163 260 109 70 450 105 162
26989-26989 107 25 25
26990-27036 310 305 254 176 119 199 277 150 260 320 138 203 210 60 38 70 672 168 15 448
27040-27080 253 468 269 299 266 191 203 145 143 72 110 226 240 561 150 506 70 150 160 40 190 210 336 180 292
27086-27096 194 344 390 331 320 450 355 60 143 120 248 168 244
27097-27187 368 415 453 385 283 282 301 286 243 288 246 239 240 600 398 1641 210 220 401 148 250 239 215 341 270 259 213 594 508 188 372 352
27197-27269 212 211 180 186 191 163 159 150 303 137 158 180 165 188 384 150 173 186 167 198 210 242 176 171 696 159 116 156 112 390 143 207
27275-27275 540 540
27278-27286 249 336 1122 605 350 371 311 377 981 590 315 524 900 315 162 793
27290-27295 300 100 53 320 193
27299-27299 211 343 333 345 286 162 160 313 225 216 488 274
27301-27310 337 360 279 286 237 241 176 347 129 210 249 426 230 188 255 128 160 75 120 75 225 90 405 450 113 224 219
27323-27365 226 323 340 316 309 251 289 246 161 488 198 234 178 412 319 113 225 300 585 450 210 480
27369-27372 149 73 105 150 630 390
27380-27499 431 468 531 391 494 370 365 438 295 297 324 267 263 302 347 251 327 235 233 122 456 316 163 188 440 539 113 253 437 357 318
27500-27566 248 301 207 241 216 198 246 164 157 136 174 142 186 162 189 175 175 318 175 159 146 138 225 118 205 40 150 120 174
27570-27570 589 440 500 278 588 90 318
27580-27580 450 150 150
27590-27598 164 210 314 132 242 170 225 294 193 146 306 254 101 157 945 273 201 243 192 132 109 90 140 183 200 170 120 381 160 255
27599-27599 220 303 295 275 218 150 315 840 360
27600-27612 156 274 240 117 225 154 220 138 145 169 222 312 132 127 73 253 105 208 277 180 300 877 90 448 227
27613-27647 209 156 256 167 173 166 228 275 177 75 315 400 210 540 90 906 90 272
27648-27648
27650-27745 195 263 242 216 233 260 280 212 364 195 206 150 128 150 200 150 400 366
27750-27848 222 216 208 200 222 180 180 196 195 102 204 219 152 92 159 366 420 146 171 298 150 59 500 343 40 105 150 60 196
27860-27860
27870-27871 201 131 200 720 130 125 488 366
27880-27889 219 255 184 197 231 175 294 166 425 299 337 177 146 295 221 152 145 341 670 120 147 188 184 132 166 77 282 193 298 101 210 227
27892-27899 195 252 270 192 160 113 115 113 220 75 100 68 270 142
28001-28035 166 173 420 130 315 225 195 210 90 194
28039-28175 199 230 113 271 170 270 600 158 540 335
28190-28193
28200-28360 180 185 263 225 296 188 150 90 190
28400-28675 208 204 186 455 158 225 105 143 217
28705-28760 212 210 135 143 810 75 360 347
28800-28825 152 89 98 149 236 144 159 137 130 132 210 135 227 138 91 148 311 181 152 114 455 113 183 50 105 80 164
28890-28899 195 90 225 225
29000-29280 112 140 150 150 58 137 73 131 270 225 149
29305-29584 203 150 195 240 128 110 180 150 162
29700-29750
29799-29799
29800- 29999 215 328 236 261 252 309 515 245 233 215 132 433 205 93 90 150 220 79 200 293 180 375 236
0 1 2 3 4 5 6 7 8 9 10 11 12 13 14 15 16 17 18 19 20 21 22 23 24 25 26 27 28 29 30 M
Length of hospital stay
500 450 400 350 300 250 200 150 100 50 0

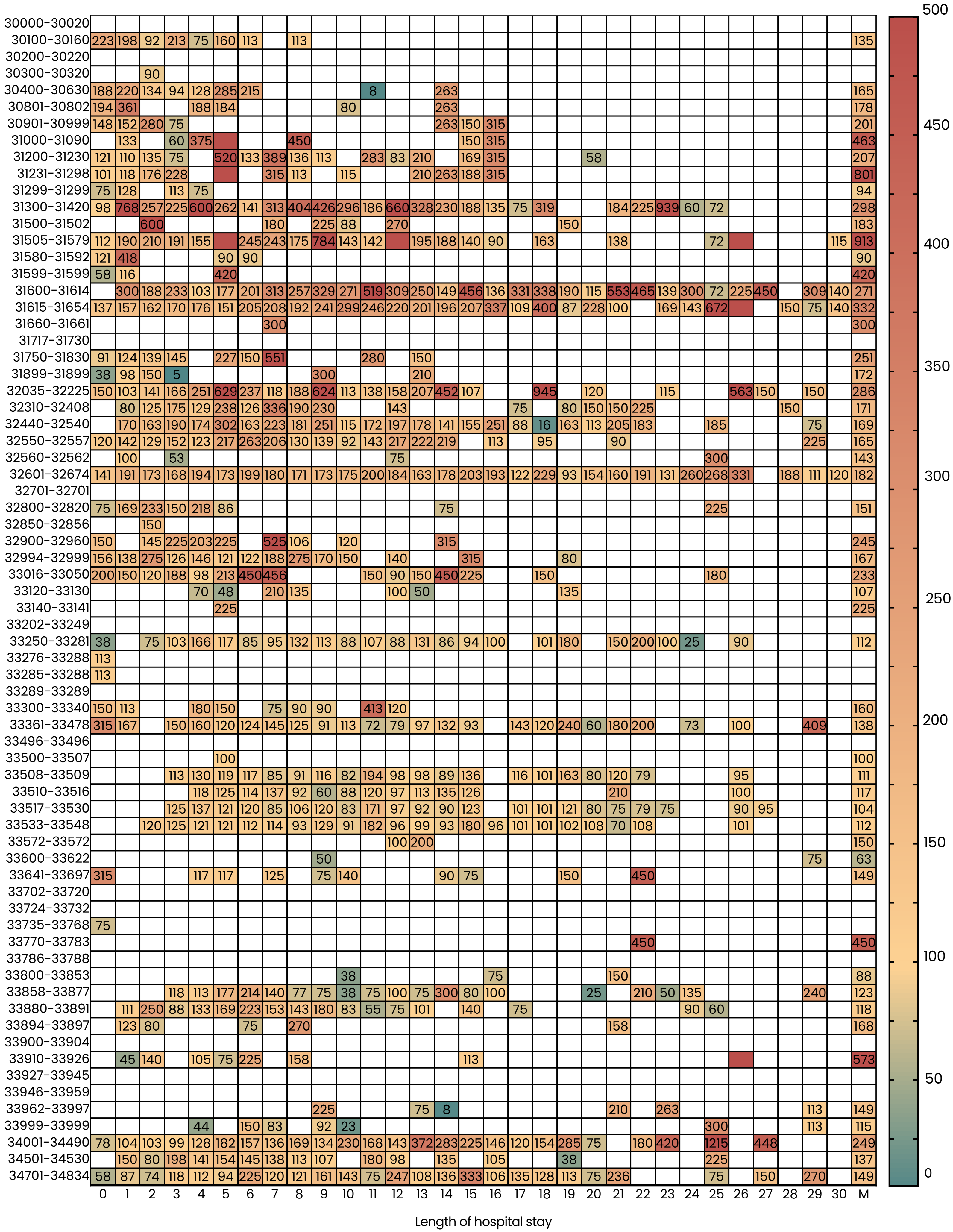
30000-30020
30100-30160
30200-30220
30300-30320
30400-30630
30801-30802
30901-30999
31000-31090
31200-31230
31231-31298
31299-31299
31300-31420
31500-31502
31505-31579
31580-31592
31599-31599
31600-31614
31615-31654
31660-31661
31717-31730
31750-31830
31899-31899
32035-32225
32310-32408
32440-32540
32550-32557
32560-32562
32601-32674
32701-32701
32800-32820
32850-32856
32900-32960
32994-32999
33016-33050
33120-33130
33140-33141
33202-33249
33250-33281
33276-33288
33285-33288
33289-33289
33300-33340
33361-33478
33496-33496
33500-33507
33508-33509
33510-33516
33517-33530
33533-33548
33572-33572
33600-33622
33641-33697
33702-33720
33724-33732
33735-33768
33770-33783
33786-33788
33800-33853
33858-33877
33880-33891
33894-33897
33900-33904
33910-33926
33927-33945
33946-33959
33962-33997
33999-33999
34001-34490
34501-34530
34701-34834
0 1 2 3 4 5 6 7 8 9 10 11 12 13 14 15 16 17 18 19 20 21 22 23 24 25 26 27 28 29 30 M
Length of hospital stay
500
450
400
350
300
250
200
150
100
50
0

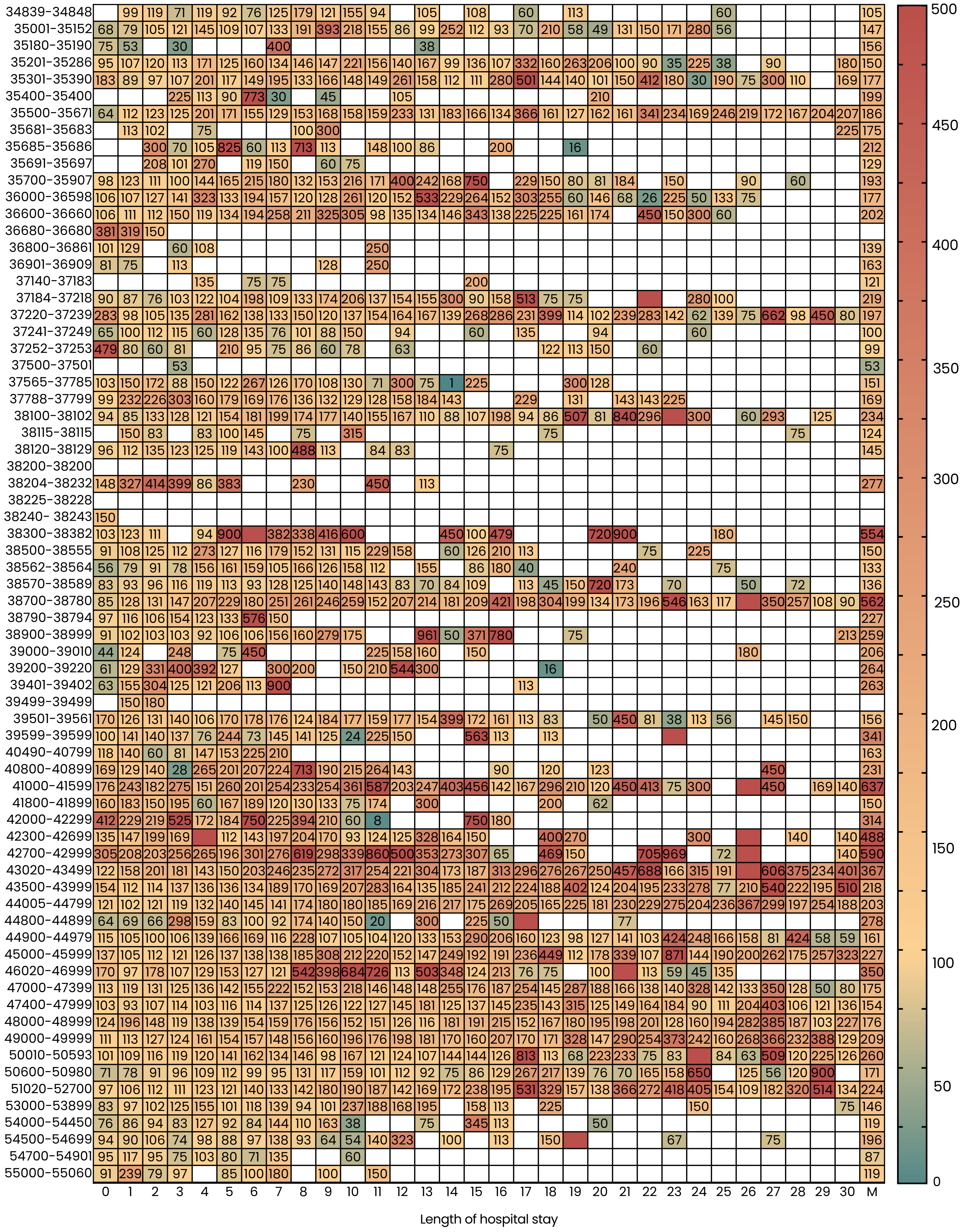
34839-34848
35001-35152
35180-35190
35201-35286
35301-35390
35400-35400
35500-35671
35681-35683
35685-35686
35691-35697
35700-35907
36000-36598
36600-36660
36680-36680
36800-36861
36901-36909
37140-37183
37184-37218
37220-37239
37241-37249
37252-37253
37500-37501
37565-37785
37788-37799
38100-38102
38115-38115
38120-38129
38200-38200
38204-38232
38225-38228
38240- 38243
38300-38382
38500-38555
38562-38564
38570-38589
38700-38780
38790-38794
38900-38999
39000-39010
39200-39220
39401-39402
39499-39499
39501-39561
39599-39599
40490-40799
40800-40899
41000-41599
41800-41899
42000-42299
42300-42699
42700-42999
43020-43499
43500-43999
44005-44799
44800-44899
44900-44979
45000-45999
46020-46999
47000-47399
47400-47999
48000-48999
49000-49999
50010-50593
50600-50980
51020-52700
53000-53899
54000-54450
54500-54699
54700-54901
55000-55060
0 1 2 3 4 5 6 7 8 9 10 11 12 13 14 15 16 17 18 19 20 21 22 23 24 25 26 27 28 29 30 M
Length of hospital stay
500
450
400
350
300
250
200
150
100
50
0

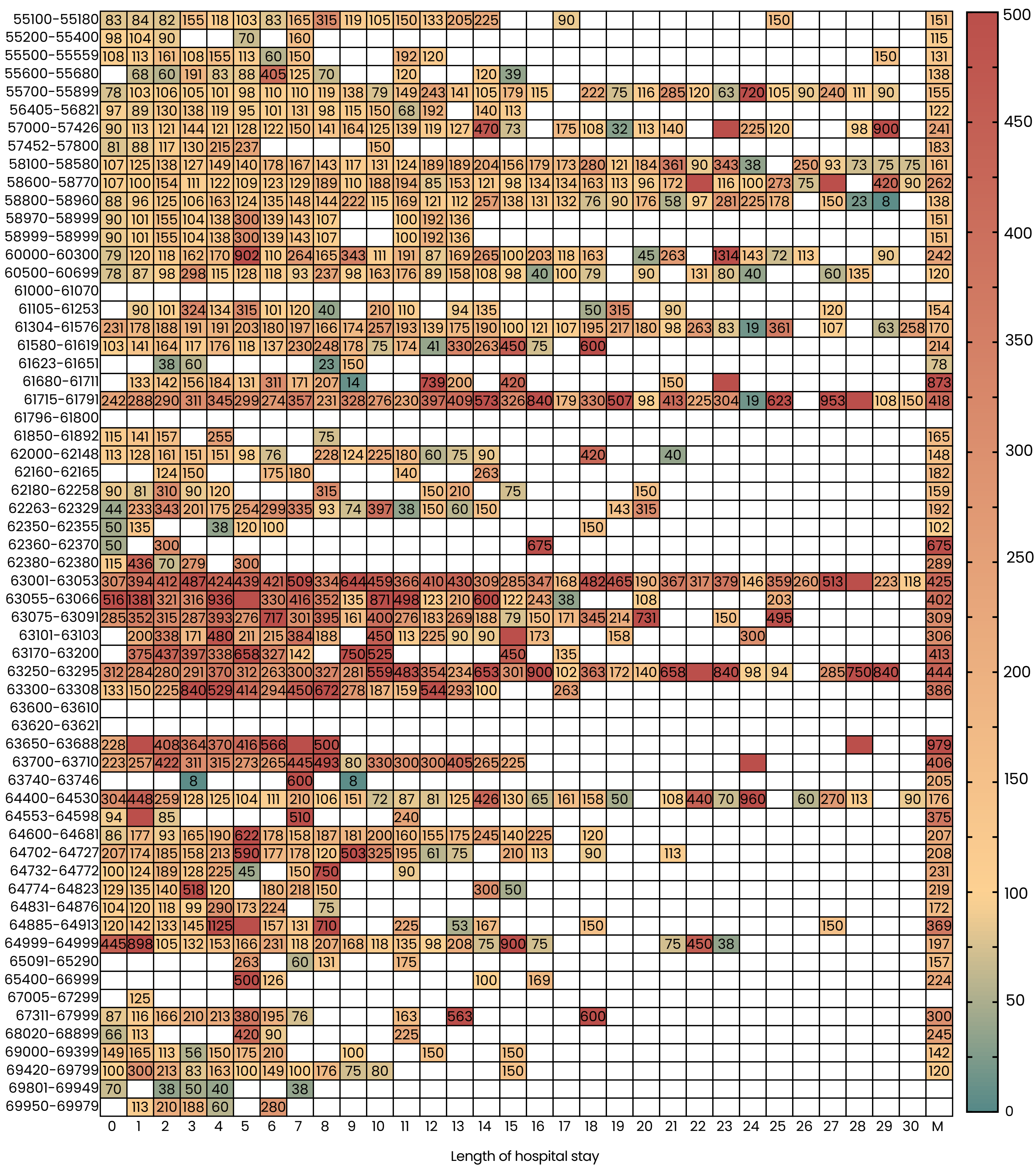
55100-55180
55200-55400
55500-55559
55600-55680
55700-55899
56405-56821
57000-57426
57452-57800
58100-58580
58600-58770
58800-58960
58970-58999
58999-58999
60000-60300
60500-60699
61000-61070
61105-61253
61304-61576
61580-61619
61623-61651
61680-61711
61715-61791
61796-61800
61850-61892
62000-62148
62160-62165
62180-62258
62263-62329
62350-62355
62360-62370
62380-62380
63001-63053
63055-63066
63075-63091
63101-63103
63170-63200
63250-63295
63300-63308
63600-63610
63620-63621
63650-63688
63700-63710
63740-63746
64400-64530
64553-64598
64600-64681
64702-64727
64732-64772
64774-64823
64831-64876
64885-64913
64999-64999
65091-65290
65400-66999
67005-67299
67311-67999
68020-68899
69000-69399
69420-69799
69801-69949
69950-69979
0 1 2 3 4 5 6 7 8 9 10 11 12 13 14 15 16 17 18 19 20 21 22 23 24 25 26 27 28 29 30 M
Length of hospital stay
500
450
400
350
300
250
200
150
100
50
0

Tables

2.1. Supplementary Table 1. Distribution of surgical procedures by Current Procedural Terminology (CPT) code range. Values represent the number and percentage of patients in each cohort (opioid vs no-opioid at discharge) who underwent each procedure.

CPT Code Range	Procedures	Opioids (n=683,828)	No Opioids (n=262,677)	% With Opioids	% Without Opioids
10004-10021	Fine Needle Aspiration Biopsy procedures	16	14	53.3	46.7
10030-10036	Introduction and Removal Procedures on the Skin, Subcutaneous and Accessory Structures	11	14	44	56
10040-10180	Incision and Drainage Procedures on the Skin, Subcutaneous and Accessory Structures	388	206	65.3	34.7
11000-11047	Debridement Procedures on the Skin	1735	885	66.2	33.8
11055-11057	Paring or Cutting Procedures on the Skin	1	0	100	0
11102-11107	Biopsy Procedures on the Skin	70	22	76.1	23.9
11200-11201	Removal of Skin Tags Procedures	122	46	72.6	27.4
11300-11313	Shaving of Epidermal or Dermal Lesions Procedures	28	19	59.6	40.4
11400-11471	Excision-Benign Lesions Procedures on the Skin	911	397	69.6	30.4
11600-11646	Excision-Malignant Lesions Procedures on the Skin	464	415	52.8	47.2
11719-11765	Surgical Procedures on the Nails	70	12	85.4	14.6
11770-11772	Surgical Procedures on the Pilonidal Cyst	48	6	88.9	11.1
11900-11983	Introduction or Removal Procedures on the Integumentary System	1601	632	71.7	28.3
12001-12021	Repair-Simple Procedures on the Integumentary System	66	21	75.9	24.1
12031-12057	Repair-Intermediate Procedures on the Integumentary System	315	92	77.4	22.6
13100-13160	Repair-Complex Procedures on the Integumentary System	808	342	70.3	29.7
14000-14350	Adjacent Tissue Transfer or Rearrangement Procedures on the Integumentary System	3793	1945	66.1	33.9
15002-15278	Skin Replacement Surgery	2208	1363	61.8	38.2
15570-15738	Flaps (Skin and/or Deep Tissues) Procedures	5466	1888	74.3	25.7
15740-15778	Other Flaps and Grafts Procedures	6998	3062	69.6	30.4
15780-15879	Other Repair (Closure) Procedures on the Integumentary System	4379	1582	73.5	26.5
15920-15999	Pressure Ulcers (Decubitus Ulcers) Procedures	32	34	48.5	51.5
16000-16036	Local Treatment Procedures for Burns	4	0	100	0
17000-17250	Destruction Procedures on Benign or Premalignant Lesions of the Integumentary System	13	6	68.4	31.6
17260-17286	Destruction Procedures on Malignant Lesions of the Integumentary System	1	1	50	50
17311-17315	Mohs Micrographic Surgery Procedures	1	2	33.3	66.7
19000-19030	Aspiration, Injection and Drainage Procedures of Breast	223	162	57.9	42.1
19081-19101	Breast Biopsy Procedures	64	31	67.4	32.6
19105-19126	Ablation, Exploration and Excision Procedures of Breast	3905	3826	50.5	49.5
19281-19298	Introduction Procedures on the Breast	431	363	54.3	45.7
19300-19307	Mastectomy Procedures	22588	12249	64.8	35.2
19316-19396	Repair and/or Reconstruction Procedures on the Breast	19532	8128	70.6	29.4
19499-19499	Other Procedures on the Breast	82	62	56.9	43.1
20100-20103	Wound Exploration-Trauma (eg, Penetrating Gunshot, Stab Wound) Procedures on the Musculoskeletal	38	17	69.1	30.9
20150-20251	General Excision Procedures on the Musculoskeletal System	253	115	68.8	31.2
20500-20705	General Introduction or Removal Procedures on the Musculoskeletal System	2684	715	79	21
20802-20838	General Replantation Procedures on the Musculoskeletal System	2	0	100	0
20900-20939	General Grafts (or Implants) Procedures on the Musculoskeletal System	16576	2932	85	15
20950-20999	Other Procedures on the Musculoskeletal System	4501	406	91.7	8.3
21010-21010	Incision Procedures on the Head	0	0	0	0
21011-21070	Excision Procedures on the Head	394	236	62.5	37.5
21073-21073	Manipulation Procedures of Head	1	0	100	0
21076-21089	Head Prosthesis Preparation	9	4	69.2	30.8

21100-21116	Introduction Procedures on the Head	2	2	50	50
21120-21296	Repair, Revision, and/or Reconstruction Procedures on the Head	883	353	71.4	28.6
21299-21299	Other Craniofacial and Maxillofacial Procedures of the Head	17	5	77.3	22.7
21315-21497	Fracture and/or Dislocation Procedures on the Head	286	106	73	27
21499-21499	Other Musculoskeletal Procedures of the Head	31	9	77.5	22.5
21501-21510	Incision Procedures on the Neck (Soft Tissues) and Thorax	112	163	40.7	59.3
21550-21630	Excision Procedures on the Neck (Soft Tissues) and Thorax	584	176	76.8	23.2
21685-21750	Repair, Revision, and/or Reconstruction Procedures on the Neck (Soft Tissues) and Thorax	172	31	84.7	15.3
21811-21825	Fracture and/or Dislocation Procedures on the Neck (Soft Tissues) and Thorax	45	27	62.5	37.5
21899-21899	Other Procedures on the Neck or Thorax	29	19	60.4	39.6
21920-21936	Excision Procedures on the Back and Flank	122	26	82.4	17.6
22010-22015	Incision Procedures on the Spine (Vertebral Column)	96	55	63.6	36.4
22100-22116	Excision Procedures on the Spine (Vertebral Column)	73	24	75.3	24.7
22206-22226	Osteotomy Procedures on the Spine (Vertebral Column)	1243	294	80.9	19.1
22310-22328	Fracture and/or Dislocation Procedures on the Spine (Vertebral Column)	276	151	64.6	35.4
22505-22505	Manipulation Procedures on the Spine (Vertebral Column)	1	1	50	50
22510-22515	Percutaneous Vertebroplasty and Vertebral Augmentation Procedures	115	31	78.8	21.2
22526-22527	Percutaneous Augmentation and Annuloplasty Procedures	2	1	66.7	33.3
22532-22819	Arthrodesis Procedures on the Spine (Vertebral Column)	25729	4905	84	16
22830-22838	Exploration Procedures on the Spine (Vertebral Column)	772	163	82.6	17.4
22840-22870	Spinal Instrumentation Procedures on the Spine (Vertebral Column)	21784	4212	83.8	16.2
22899-22899	Other Procedures on the Spine (Vertebral Column)	99	21	82.5	17.5
22900-22905	Excision Procedures on the Abdomen	372	128	74.4	25.6
22999-22999	Other Procedures on the Abdomen	182	56	76.5	23.5
23000-23044	Incision Procedures on the Shoulder	88	57	60.7	39.3
23065-23220	Excision Procedures on the Shoulder	781	167	82.4	17.6
23330-23350	Introduction or Removal Procedures on the Shoulder	20	15	57.1	42.9
23395-23491	Repair, Revision, and/or Reconstruction Procedures on the Shoulder	9394	1549	85.8	14.2
23500-23680	Fracture and/or Dislocation Procedures on the Shoulder	2100	460	82	18
23700-23700	Manipulation Procedures on the Shoulder	40	11	78.4	21.6
23800-23802	Arthrodesis Procedures on the Shoulder	12	0	100	0
23900-23921	Amputation Procedures on the Shoulder	6	3	66.7	33.3
23929-23929	Other Procedures on the Shoulder	44	6	88	12
23930-24006	Incision Procedures on the Humerus (Upper Arm) and Elbow	78	33	70.3	29.7
24065-24155	Excision Procedures on the Humerus (Upper Arm) and Elbow	489	218	69.2	30.8
24160-24220	Introduction or Removal Procedures on the Humerus (Upper Arm) and Elbow	17	13	56.7	43.3
24300-24498	Repair, Revision, and/or Reconstruction Procedures on the Humerus (Upper Arm) and Elbow	1999	514	79.5	20.5
24500-24685	Fracture and/or Dislocation Procedures on the Humerus (Upper Arm) and Elbow	1863	477	79.6	20.4
24800-24802	Arthrodesis Procedures on the Humerus (Upper Arm) and Elbow	0	0	0	0
24900-24940	Amputation Procedures on the Humerus (Upper Arm) and Elbow	10	3	76.9	23.1
24999-24999	Other Procedures on the Humerus or Elbow	43	4	91.5	8.5
25000-25040	Incision Procedures on the Forearm and Wrist	423	279	60.3	39.7
25065-25240	Excision Procedures on the Forearm and Wrist	1522	600	71.7	28.3
25246-25259	Introduction or Removal Procedures on the Forearm and Wrist	28	12	70	30
25260-25492	Repair, Revision, and/or Reconstruction Procedures on the Forearm and Wrist	2142	524	80.3	19.7
25500-25695	Fracture and/or Dislocation Procedures on the Forearm and Wrist	4409	1099	80	20
25800-25830	Arthrodesis Procedures on the Forearm and Wrist	44	7	86.3	13.7
25900-25931	Amputation Procedures on the Forearm and Wrist	14	9	60.9	39.1
25999-25999	Other Procedures on the Forearm or Wrist	13	2	86.7	13.3
26010-26080	Incision Procedures on the Hand and Fingers	162	76	68.1	31.9
26100-26262	Excision Procedures on the Hand and Fingers	145	42	77.5	22.5

26320-26320	Introduction or Removal Procedures on the Hand and Fingers	9	2	81.8	18.2
26340-26596	Repair, Revision, and/or Reconstruction Procedures on the Hand and Fingers	1973	666	74.8	25.2
26600-26785	Fracture and/or Dislocation Procedures on the Hand and Fingers	1672	515	76.5	23.5
26820-26863	Arthrodesis Procedures on the Hand and Fingers	80	17	82.5	17.5
26910-26952	Amputation Procedures on the Hand and Fingers	416	187	69	31
26989-26989	Other Procedures on the Hands or Fingers	23	20	53.5	46.5
26990-27036	Incision Procedures on the Pelvis and Hip Joint	244	79	75.5	24.5
27040-27080	Excision Procedures on the Pelvis and Hip Joint	749	262	74.1	25.9
27086-27096	Introduction or Removal Procedures on the Pelvis and Hip Joint	42	20	67.7	32.3
27097-27187	Repair, Revision, and/or Reconstruction Procedures on the Pelvis and Hip Joint	42101	6031	87.5	12.5
27197-27269	Fracture and/or Dislocation Procedures on the Pelvis and Hip Joint	12486	7555	62.3	37.7
27275-27275	Manipulation Procedures on the Pelvis and Hip Joint	1	2	33.3	66.7
27278-27286	Arthrodesis Procedures on the Pelvis and Hip Joint	273	86	76	24
27290-27295	Amputation Procedures on the Pelvis and Hip Joint	13	8	61.9	38.1
27299-27299	Other Procedures on the Pelvis or Hip Joint	305	40	88.4	11.6
27301-27310	Incision Procedures on the Femur (Thigh Region) and Knee Joint	468	203	69.7	30.3
27323-27365	Excision Procedures on the Femur (Thigh Region) and Knee Joint	1249	348	78.2	21.8
27369-27372	Introduction or Removal Procedures on the Femur (Thigh Region) and Knee Joint	29	19	60.4	39.6
27380-27499	Repair, Revision, and/or Reconstruction Procedures on the Femur (Thigh Region) and Knee Joint	64602	6940	90.3	9.7
27500-27566	Fracture and/or Dislocation Procedures on the Femur (Thigh Region) and Knee Joint	3166	1109	74.1	25.9
27570-27570	Manipulation Procedures on the Femur (Thigh Region) and Knee Joint	90	21	81.1	18.9
27580-27580	Arthrodesis Procedures on the Femur (Thigh Region) and Knee Joint	3	3	50	50
27590-27598	Amputation Procedures on the Femur (Thigh Region) and Knee Joint	532	433	55.1	44.9
27599-27599	Other Procedures on the Femur or Knee Joint	177	33	84.3	15.7
27600-27612	Incision Procedures on the Leg (Tibia and Fibula) and Ankle Joint	419	181	69.8	30.2
27613-27647	Excision Procedures on the Leg (Tibia and Fibula) and Ankle Joint	514	172	74.9	25.1
27648-27648	Injection Procedures on the Leg (Tibia and Fibula) and Ankle Joint	0	0	0	0
27650-27745	Repair, Revision, and/or Reconstruction Procedures on the Leg (Tibia and Fibula) and Ankle Joint	2804	660	80.9	19.1
27750-27848	Fracture and/or Dislocation Procedures on the Leg (Tibia and Fibula) and Ankle Joint	6639	1503	81.5	18.5
27860-27860	Manipulation Procedures on the Leg (Tibia and Fibula) and Ankle Joint	0	2	0	100
27870-27871	Arthrodesis Procedures on the Leg (Tibia and Fibula) and Ankle Joint	36	3	92.3	7.7
27880-27889	Amputation Procedures on the Leg (Tibia and Fibula) and Ankle Joint	1134	918	55.3	44.7
27892-27899	Other Procedures on the Leg (Tibia and Fibula) and Ankle Joint	72	28	72	28
28001-28035	Incision Procedures on the Foot and Toes	43	19	69.4	30.6
28039-28175	Excision Procedures on the Foot and Toes	268	79	77.2	22.8
28190-28193	Removal of Foreign Body Procedures on the Foot and Toes	0	2	0	100
28200-28360	Repair, Revision, and/or Reconstruction Procedures on the Foot and Toes	222	54	80.4	19.6
28400-28675	Fracture and/or Dislocation Procedures on the Foot and Toes	153	42	78.5	21.5
28705-28760	Arthrodesis Procedures on the Foot and Toes	130	24	84.4	15.6
28800-28825	Amputation Procedures on the Foot and Toes	413	438	48.5	51.5
28890-28899	Other Procedures on the Foot and Toes	6	2	75	25
29000-29280	Body and Upper Extremity Application of Casts and Strapping	79	33	70.5	29.5
29305-29584	Lower Extremity Application of Casts and Strapping	80	17	82.5	17.5
29700-29750	Removal or Repair of Casts and Strapping	0	0	0	0
29799-29799	Other Casting or Strapping Procedures	0	0	0	0
29800- 29999	Endoscopy/Arthroscopy Procedures on the Musculoskeletal System	24400	5269	82.2	17.8
30000-30020	Incision Procedures on the Nose	0	0	0	0
30100-30160	Excision Procedures on the Nose	215	52	80.5	19.5
30200-30220	Introduction Procedures on the Nose	0	6	0	100
30300-30320	Removal of Foreign Body Procedures on the Nose	1	0	100	0
30400-30630	Repair Procedures on the Nose	309	74	80.7	19.3

30801-30802	Destruction Procedures on the Nose	53	15	77.9	22.1
30901-30999	Other Procedures on the Nose	83	12	87.4	12.6
31000-31090	Incision Procedures on the Accessory Sinuses	18	11	62.1	37.9
31200-31230	Excision Procedures on the Accessory Sinuses	91	72	55.8	44.2
31231-31298	Endoscopy Procedures on the Accessory Sinuses	106	58	64.6	35.4
31299-31299	Other Procedures on the Accessory Sinuses	11	2	84.6	15.4
31300-31420	Excision Procedures on the Larynx	228	120	65.5	34.5
31500-31502	Introduction Procedures on the Larynx	13	5	72.2	27.8
31505-31579	Endoscopy Procedures on the Larynx	515	184	73.7	26.3
31580-31592	Repair Procedures on the Larynx	67	79	45.9	54.1
31599-31599	Other Procedures on the Larynx	82	37	68.9	31.1
31600-31614	Incision Procedures on the Trachea and Bronchi	479	261	64.7	35.3
31615-31654	Endoscopy Procedures on the Trachea and Bronchi	3084	744	80.6	19.4
31660-31661	Thermoplasty Procedures on the Trachea and Bronchi	1	1	50	50
31717-31730	Introduction Procedures on the Trachea and Bronchi	0	0	0	0
31750-31830	Excision and Repair Procedures on the Trachea and Bronchi	69	48	59	41
31899-31899	Other Procedures on the Trachea or Bronchi	28	17	62.2	37.8
32035-32225	Incision Procedures on the Lungs and Pleura	284	96	74.7	25.3
32310-32408	Excision/Resection Procedures on the Lungs and Pleura	69	17	80.2	19.8
32440-32540	Removal Procedures on the Lungs and Pleura	959	253	79.1	20.9
32550-32557	Introduction and Removal Procedures on the Lungs and Pleura	275	116	70.3	29.7
32560-32562	Destruction Procedures on the Lungs and Pleura	20	18	52.6	47.4
32601-32674	Thoracoscopy (Video-assisted thoracic surgery [VATS]) on the Lungs and Pleura	8317	2133	79.6	20.4
32701-32701	Stereotactic Radiation Therapy Procedures on the Lungs and Pleura	0	0	0	0
32800-32820	Repair Procedures on the Lungs and Pleura	29	6	82.9	17.1
32850-32856	Lung Transplantation Procedures	2	1	66.7	33.3
32900-32960	Surgical Collapse Therapy Procedures on the Lungs and Pleura	28	43	39.4	60.6
32994-32999	Other Procedures on the Lungs and Pleura	85	18	82.5	17.5
33016-33050	Surgical Procedures on the Pericardium	47	61	43.5	56.5
33120-33130	Excision Procedures of Cardiac Tumor	15	22	40.5	59.5
33140-33141	Transmyocardial Revascularization Procedures	1	0	100	0
33202-33249	Pacemaker or Implantable Defibrillator Procedures	3	11	21.4	78.6
33250-33281	Electrophysiologic Operative Procedures on the Heart and Pericardium	155	184	45.7	54.3
33276-33288	Phrenic Nerve Stimulation System	4	4	50	50
33285-33288	Introduction or Removal of Subcutaneous Cardiac Rhythm Monitor	4	4	50	50
33289-33289	Implantation of Hemodynamic Monitor	1	0	100	0
33300-33340	Surgical Procedures on the Heart (Including Valves) and Great Vessels	15	18	45.5	54.5
33361-33478	Surgical Procedures on Cardiac Valves	314	893	26	74
33496-33496	Other Cardiac Valvular Procedures	0	0	0	0
33500-33507	Coronary Artery Anomaly Procedures	2	11	15.4	84.6
33508-33509	Endoscopy Procedures on the Heart and Pericardium	398	442	47.4	52.6
33510-33516	Venous Grafting Only for Coronary Artery Bypass	57	150	27.5	72.5
33517-33530	Combined Arterial-Venous Grafting for Coronary Bypass	525	906	36.7	63.3
33533-33548	Arterial Grafting for Coronary Artery Bypass	685	1113	38.1	61.9
33572-33572	Coronary Endarterectomy Procedures	7	16	30.4	69.6
33600-33622	Repair Procedures for Single Ventricle and Other Complex Cardiac Anomalies	2	2	50	50
33641-33697	Repair Procedures for Septal Defect	17	39	30.4	69.6
33702-33720	Repair Procedures for the Sinus of Valsalva	0	2	0	100
33724-33732	Repair Procedures for Venous Anomalies	0	0	0	0
33735-33768	Shunting Procedures on the Heart and Pericardium	1	0	100	0
33770-33783	Repair Procedures for Transposition of the Great Vessels	2	0	100	0

33786-33788	Repair Procedures for Truncus Arteriosus	0	0	0	0
33800-33853	Repair Procedures for Aortic Anomalies	3	2	60	40
33858-33877	Repair Procedures for Thoracic Aortic Aneurysm	73	171	29.9	70.1
33880-33891	Endovascular Repair Procedures of the Descending Thoracic Aorta	150	252	37.3	62.7
33894-33897	Endovascular Repair of Congenital Heart and Vascular Defects	13	16	44.8	55.2
33900-33904	Endovascular Repair of Pulmonary Artery	0	1	0	100
33910-33926	Surgical Procedures on the Pulmonary Artery	12	27	30.8	69.2
33927-33945	Heart/Lung Transplantation Procedures	0	0	0	0
33946-33959	Extracorporeal Membrane Oxygenation or Extracorporeal Life Support Services and Procedures	3	3	50	50
33962-33997	Cardiac Assist Procedures	9	18	33.3	66.7
33999-33999	Other Cardiac Surgery Procedures	20	125	13.8	86.2
34001-34490	Embolectomy/Thrombectomy Procedures on Arteries and Veins	505	530	48.8	51.2
34501-34530	Venous Reconstruction Procedures	101	31	76.5	23.5
34701-34834	Endovascular Repair Procedures of the Abdominal Aorta and/or Iliac Arteries	1030	1897	35.2	64.8
34839-34848	Fenestrated Endovascular Repair Procedures of the Visceral and Infrarenal Aorta	154	235	39.6	60.4
35001-35152	Direct Repair of Aneurysm or Excision (Partial or Total) and Graft Insertion for Aneurysm, Pseudoaneurysm,	666	487	57.8	42.2
35180-35190	Arteriovenous Fistula Repair Procedures	10	17	37	63
35201-35286	Repair Procedures Blood Vessel Other Than for Fistula, With or Without Patch Angioplasty	1043	443	70.2	29.8
35301-35390	Thromboendarterectomy Procedures on Arteries and Veins	3502	3634	49.1	50.9
35400-35400	Angioscopy Procedures on Arteries and Veins	10	2	83.3	16.7
35500-35671	Bypass Graft Procedures	2557	1482	63.3	36.7
35681-35683	Composite Graft Procedures on Arteries and Veins	32	17	65.3	34.7
35685-35686	Adjuvant Techniques Procedures on Arteries and Veins	33	16	67.3	32.7
35691-35697	Arterial Transposition Procedures	29	13	69	31
35700-35907	Repair, Excision, Exploration, Revision Procedures on Arteries and Veins	433	323	57.3	42.7
36000-36598	Vascular Introduction and Injection Procedures	2239	2183	50.6	49.4
36600-36660	Arterial Procedures	366	181	66.9	33.1
36680-36680	Intraosseous Procedures on Arteries and Veins	18	1	94.7	5.3
36800-36861	Hemodialysis Access, Intervascular Cannulation for Extracorporeal Circulation, or Shunt Insertion Procedures	39	64	37.9	62.1
36901-36909	Dialysis Circuit Procedures	12	52	18.8	81.2
37140-37183	Portal Decompression Procedures on Arteries and Veins	9	4	69.2	30.8
37184-37218	Transcatheter Procedures on Arteries and Veins	551	872	38.7	61.3
37220-37239	Endovascular Revascularization	2216	5003	30.7	69.3
37241-37249	Vascular Embolization and Occlusion Procedures on Arteries and Veins	143	304	32	68
37252-37253	Intravascular Ultrasound Procedures on Arteries and Veins	91	196	31.7	68.3
37500-37501	Vascular Endoscopy Procedures	2	0	100	0
37565-37785	Ligation Procedures on Arteries and Veins	687	829	45.3	54.7
37788-37799	Other Artery and Vein Procedures	448	372	54.6	45.4
38100-38102	Excision Procedures on the Spleen	536	246	68.5	31.5
38115-38115	Repair Procedures on the Spleen	19	11	63.3	36.7
38120-38129	Laparoscopic Procedures on the Spleen	399	123	76.4	23.6
38200-38200	Injection Procedure on the Spleen	1	0	100	0
38204-38232	Bone Marrow or Stem Cell Services/Procedures	108	36	75	25
38225-38228	Cellular and Gene therapy Procedures	0	0	0	0
38240- 38243	Surgical Procedures on the Hemic and Lymphatic Systems	1	1	50	50
38300-38382	Incision Procedures on the Lymph Nodes and Lymphatic Channels	84	18	82.4	17.6
38500-38555	Excision Procedures on the Lymph Nodes and Lymphatic Channels	12137	5692	68.1	31.9
38562-38564	Limited Lymphadenectomy for Staging Procedures	496	118	80.8	19.2
38570-38589	Laparoscopic Procedures on the Lymph Nodes and Lymphatic Channels	11493	3168	78.4	21.6
38700-38780	Radical Lymphadenectomy (Radical Resection of Lymph Nodes)	6037	2499	70.7	29.3
38790-38794	Introduction Procedures on the Lymph Nodes and Lymphatic Channels	1630	558	74.5	25.5

38900-38999	Other Procedures of the Hemic or Lymphatic System	7357	3008	71	29
39000-39010	Incision Procedures on the Mediastinum	25	16	61	39
39200-39220	Excision/Resection Procedures on the Mediastinum	123	33	78.8	21.2
39401-39402	Endoscopy Procedures on the Mediastinum	95	34	73.6	26.4
39499-39499	Other Procedures on the Mediastinum	5	3	62.5	37.5
39501-39561	Repair Procedures on the Diaphragm	474	128	78.7	21.3
39599-39599	Other Procedures on the Diaphragm	123	21	85.4	14.6
40490-40799	Surgical Procedures on the Lips	128	79	61.8	38.2
40800-40899	Surgical Procedures on the Vestibule of Mouth	242	152	61.4	38.6
41000-41599	Surgical Procedures on the Tongue and Floor of Mouth	957	474	66.9	33.1
41800-41899	Surgical Procedures on the Dentoalveolar Structures	135	89	60.3	39.7
42000-42299	Surgical Procedures on the Palate and Uvula	370	158	70.1	29.9
42300-42699	Surgical Procedures on the Salivary Gland and Ducts	1809	1034	63.6	36.4
42700-42999	Surgical Procedures on the Pharynx, Adenoids, and Tonsils	3659	1100	76.9	23.1
43020-43499	Surgical Procedures on the Esophagus	10108	3575	73.9	26.1
43500-43999	Surgical Procedures on the Stomach	8783	5577	61.2	38.8
44005-44799	Surgical Procedures on the Intestines (Except Rectum)	64167	31837	66.8	33.2
44800-44899	Surgical Procedures on Meckel's Diverticulum and the Mesentery	223	120	65	35
44900-44979	Surgical Procedures on the Appendix	39004	14523	72.9	27.1
45000-45999	Surgical Procedures on the Colon and Rectum	9541	4146	69.7	30.3
46020-46999	Surgical Procedures on the Anus	2618	1094	70.5	29.5
47000-47399	Surgical Procedures on the Liver	8195	2494	76.7	23.3
47400-47999	Surgical Procedures on the Biliary Tract	49518	16699	74.8	25.2
48000-48999	Surgical Procedures on the Pancreas	7621	2472	75.5	24.5
49000-49999	Surgical Procedures on the Abdomen, Peritoneum, and Omentum	93245	30654	75.3	24.7
50010-50593	Surgical Procedures on the Kidney	11067	3123	78	22
50600-50980	Surgical Procedures on the Ureter	2765	1054	72.4	27.6
51020-52700	Surgical Procedures on the Bladder	31237	24831	55.7	44.3
53000-53899	Surgical Procedures on the Urethra	1478	1072	58	42
54000-54450	Surgical Procedures on the Penis	528	281	65.3	34.7
54500-54699	Surgical Procedures on the Testis	1397	747	65.2	34.8
54700-54901	Surgical Procedures on the Epididymis	492	244	66.8	33.2
55000-55060	Surgical Procedures on the Tunica Vaginalis	1149	628	64.7	35.3
55100-55180	Surgical Procedures on the Scrotum	440	348	55.8	44.2
55200-55400	Surgical Procedures on the Vas Deferens	125	48	72.3	27.7
55500-55559	Surgical Procedures on the Spermatic Cord	604	217	73.6	26.4
55600-55680	Surgical Procedures on the Seminal Vesicles	129	31	80.6	19.4
55700-55899	Surgical Procedures on the Prostate	10866	4635	70.1	29.9
56405-56821	Surgical Procedures on the Vulva, Perineum and Introitus	1600	909	63.8	36.2
57000-57426	Surgical Procedures on the Vagina	12975	5696	69.5	30.5
57452-57800	Surgical Procedures on the Cervix Uteri	283	142	66.6	33.4
58100-58580	Surgical Procedures on the Corpus Uteri	56078	16429	77.3	22.7
58600-58770	Surgical Procedures on the Oviduct/Ovary	20083	7603	72.5	27.5
58800-58960	Surgical Procedures on the Ovary	3216	1117	74.2	25.8
58970-58999	Surgical Procedures for In Vitro Fertilization	205	63	76.5	23.5
58999-58999	Other Procedures on the Female Genital System	204	63	76.4	23.6
60000-60300	Surgical Procedures on the Thyroid Gland	11066	7738	58.8	41.2
60500-60699	Surgical Procedures on the Parathyroid, Thymus, Adrenal Glands, Pancreas, and Carotid Body	6104	3848	61.3	38.7
61000-61070	Injection, Drainage, or Aspiration Procedures on the Skull, Meninges, and Brain	1	0	100	0
61105-61253	Twist Drill, Burr Hole(s), or Trephine Procedures on the Skull, Meninges, and Brain	59	101	36.9	63.1
61304-61576	Craniectomy or Craniotomy Procedures	4672	3957	54.1	45.9

61580-61619	Skull Base Surgical Procedures	281	182	60.7	39.3
61623-61651	Endovascular Therapy Procedures on the Skull, Meninges, and Brain	7	9	43.8	56.2
61680-61711	Surgery for Aneurysm, Arteriovenous Malformation or Vascular Disease Procedures on the Skull, Meninges,	207	195	51.5	48.5
61715-61791	Stereotaxis Procedures on the Skull, Meninges, and Brain	5267	2137	71.1	28.9
61796-61800	Stereotactic Radiosurgery (Cranial) Procedures on the Skull, Meninges, and Brain	1	1	50	50
61850-61892	Neurostimulators (Intracranial) Procedures on the Skull, Meninges, and Brain	393	370	51.5	48.5
62000-62148	Repair Procedures on the Skull, Meninges, and Brain	236	116	67	33
62160-62165	Neuroendoscopy Procedures on the Skull, Meninges, and Brain	31	14	68.9	31.1
62180-62258	Cerebrospinal Fluid (CSF) Shunt Procedures	27	21	56.2	43.8
62263-62329	Injection, Drainage, or Aspiration Procedures on the Spine and Spinal Cord	249	83	75	25
62350-62355	Catheter Implantation Procedures on the Spine and Spinal Cord	12	4	75	25
62360-62370	Reservoir/Pump Implantation Procedures on the Spine and Spinal Cord	8	5	61.5	38.5
62380-62380	Endoscopic Decompression of Neural Elements and/or Excision of Herniated Intervertebral Discs	58	17	77.3	22.7
63001-63053	Posterior Extradural Laminotomy or Laminectomy for Exploration/ Decompression of Neural Elements or	24637	4616	84.2	15.8
63055-63066	Transpedicular or Costovertebral Approach for Posterolateral Extradural Exploration/Decompression	508	108	82.5	17.5
63075-63091	Anterior or Anterolateral Approach for Extradural Exploration/Decompression Procedures on the Spine and	772	211	78.5	21.5
63101-63103	Lateral Extracavitary Approach for Extradural Exploration/Decompression Procedures on the Spine and	83	33	71.6	28.4
63170-63200	Incision Procedures on the Spine and Spinal Cord	125	24	83.9	16.1
63250-63295	Excision by Laminectomy of Lesion Other Than Herniated Disk Procedures	2070	586	77.9	22.1
63300-63308	Excision, Anterior or Anterolateral Approach, Intraspinal Lesion Procedures on the Spine and Spinal Cord	75	34	68.8	31.2
63600-63610	Stereotaxis Procedures on the Spine and Spinal Cord	0	0	0	0
63620-63621	Stereotactic Radiosurgery (Spinal) Procedures on the Spine and Spinal Cord	0	0	0	0
63650-63688	Neurostimulators (Spinal) Procedures	79	22	78.2	21.8
63700-63710	Repair Procedures on the Spine and Spinal Cord	237	76	75.7	24.3
63740-63746	Shunt, Spinal CSF Procedures	4	5	44.4	55.6
64400-64530	Introduction/Injection of Anesthetic Agent (Nerve Block), Diagnostic or Therapeutic Procedures on the	6380	1291	83.2	16.8
64553-64598	Neurostimulator Procedures on the Peripheral Nerves	27	9	75	25
64600-64681	Destruction by Neurolytic Agent (eg, Chemical, Thermal, Electrical or Radiofrequency) and	318	103	75.5	24.5
64702-64727	Neuroplasty (Exploration, Neurolysis or Nerve Decompression) Procedures on the Extracranial Nerves,	1628	501	76.5	23.5
64732-64772	Transection or Avulsion Procedures on the Extracranial Nerves, Peripheral Nerves, and Autonomic Nervous	207	39	84.1	15.9
64774-64823	Excision Procedures on the Extracranial Nerves, Peripheral Nerves, and Autonomic Nervous System	99	20	83.2	16.8
64831-64876	Neurorrhaphy Procedures	263	95	73.5	26.5
64885-64913	Neurorrhaphy With Nerve Graft, Vein Graft or Conduit Procedures	612	293	67.6	32.4
64999-64999	Other Procedures of the Nervous System	854	175	83	17
65091-65290	Surgical Procedures on the Eyeball	11	4	73.3	26.7
65400-66999	Surgical Procedures on the Anterior Segment of the Eye	7	2	77.8	22.2
67005-67299	Surgical Procedures on the Posterior Segment of the Eye	1	0	100	0
67311-67999	Surgical Procedures on the Ocular Adnexa	186	65	74.1	25.9
68020-68899	Surgical Procedures on the Conjunctiva	17	5	77.3	22.7
69000-69399	Surgical Procedures on the External Ear	82	48	63.1	36.9
69420-69799	Surgical Procedures on the Middle Ear	1011	898	53	47
69801-69949	Surgical Procedures on the Inner Ear	14	6	70	30
69950-69979	Surgical Procedures on the Temporal Bone, Middle Fossa Approach	8	8	50	50

2.2. Supplementary Table 2. Postoperative characteristics of patients discharge with and without opioids. Data are reported as n (%), unless otherwise specified. Cramér’s V is provided to quantify the magnitude of association between opioid prescribing status and each outcome. Statistical significance was defined as $p < 0.05$.

`	**Total patients (n=945,505)**	**No opioids (n=261,677)**	**Opioids (n=683,828)**	**p-value**	**Cramér's V**
**Length of stay mean days (SD)**	2.4 (4.0)	3.3 (5.1)	2.0 (3.4)	**<0.0001**	0.30
**Any readmission**	44,827 (4.7)	14,695 (5.6)	30,132 (4.4)	**<0.0001**	0.03
**Unplanned readmission**	43,554 (4.6)	14,096 (5.4)	29,458 (4.3)	**<0.0001**	0.02
**Any major complication**	81,542 (8.6)	28,825 (11)	52,717 (7.7)	**<0.0001**	0.05
Superficial infection	16,167 (1.7)	4504 (1.7)	11,663 (1.7)	**0.61**	<0.001
Deep wound infection	2537 (0.3)	850 (0.3)	1687 (0.3)	**<0.0001**	0.007
Organ space infection	15,442 (1.6)	4892 (1.9)	10,550 (1.5)	**<0.0001**	0.01
Dehiscence	3193 (0.3)	1022 (0.4)	2171 (0.3)	**<0.0001**	0.006
Bleeding requiring transfusion	30,527 (3.2)	11,489 (4.4)	19,038 (2.8)	**<0.0001**	0.04
Pneumonia	6367 (0.7)	3013 (1.2)	3354 (0.5)	**<0.0001**	0.04
Unplanned reintubation	1837 (0.2)	976 (0.4)	861 (0.1)	**<0.0001**	0.03
Ventilator use > 48 hours	2514 (0.3)	1483 (0.6)	1031 (0.2)	**<0.0001**	0.04
Pulmonary embolism	2909 (0.3)	1001 (0.4)	1908 (0.3)	**<0.0001**	0.008
Renal insufficiency	4441 (0.5)	1853 (0.7)	2588 (0.4)	**<0.0001**	0.02
Renal failure	641 (0.1)	365 (0.1)	276 (0.0)	**<0.0001**	0.02
Stroke	1065 (0.1)	571 (0.2)	494 (0.1)	**<0.0001**	0.02
Cardiac arrest	454 (0.0)	247 (0.1)	207 (0.0)	**<0.0001**	0.01
Myocardial infarction	2401 (0.3)	1073 (0.4)	1328 (0.2)	**<0.0001**	0.02
DVT	4360 (0.5)	1495 (0.6)	2865 (0.4)	**<0.0001**	0.01
Sepsis	5948 (0.6)	2548 (1.0)	3400 (0.5)	**<0.0001**	0.03
Septic shock	4060 (0.4)	2013 (0.8)	2047 (0.3)	**<0.0001**	0.03
C. diff. infection	1774 (0.2)	697 (0.3)	1077 (0.2)	**<0.0001**	0.01
**Delirium**					
Screened for Delirium	63,626 (6.7)	22,845 (8.7)	40,781 (6.0)	**<0.0001**	0.01
Screening tool detected delirium	5356 (0.57)	2780 (1.1)	2576 (0.4)	**<0.0001**	0.10
Delirium clinically diagnosed	9555 (1.0)	5020 (1.9)	4535 (0.7)	**<0.0001**	0.09
**Discharge Destination**				**<0.0001**	0.10
Home/Permanent residence	894,353 (95)	239,204 (91)	655,149 (96)		
Other Facility	47,835 (5.1)	20,139 (7.7)	27,696 (4.1)		
Against Medical advice (AMA)	1163 (0.1)	807 (0.3)	356 (0.1)		
Unknown	1928 (0.2)	1463 (0.6)	465 (0.1)		
Acute Care Hospital	226 (0.0)	64 (0.0)	162 (0.0)		
**Discharged home with services**	33,952 (3.6)	10,415 (4.0)	23,537 (3.4)	**<0.0001**	0.05
**Transfer to higher level of care**	350 (0.0)	247 (0.1)	103 (0.0)	**0.01**	0.06
**End of life/Withdrawal of care**	1172 (0.1)	618 (0.2)	554 (0.1)	**<0.0001**	0.02
**COVID-19 Diagnosis**	3747 (0.4)	1292 (0.5)	2455 (0.4)	**<0.0001**	0.01
**COVID-related complication**	4441 (0.5)	1653 (0.6)	2788 (0.4)	**<0.0001**	0.02
**Supplemental oxygen therapy**	15,387 (1.6)	6311 (2.4)	9076 (1.3)	**<0.0001**	0.04
**Discharge Functional Status**				**<0.0001**	0.05
Independent	95,034 (10)	34,647 (13)	60,387 (8.8)		
Partially Dependent	46,855 (5.0)	16,806 (6.4)	30,049 (4.4)		
Totally Dependent	2839 (0.3)	1521 (0.6)	1318 (0.2)		
Unknown	9433 (1.0)	3519 (1.3)	5914 (0.9)		
Expired	14 (0.0)	7 (0.0)	7 (0.0)		
**Functional Status Decline**	40,826 (4.3)	13,636 (5.2)	27,190 (4.0)	**<0.0001**	0.03

2.3. Supplementary Table 3. Comparison of included and excluded patients based on availability of post-discharge opioid prescribing data or prolonged hospitalization (>30 days). Baseline demographic characteristics, comorbidities, perioperative factors, and postoperative outcomes are shown. Continuous variables are presented as mean (standard deviation) and compared using Welch's t-tests; categorical variables are presented as counts (percentages) and compared using χ^2 tests. Effect sizes are reported as Cohen's d for continuous variables and Cramér's V for categorical variables.

**Continuous variables**	**Total (n=963,565)**	**Excluded (n=18,060)**	**Included (n=945,505)**	**p-value**	**Cohen's d**
Age mean years (SD)	57 (17)	67 (15)	57 (17)	<0.001	0.64
BMI mean kg/m^2 (SD)	30 (7.1)	28 (8.0)	30 (7.1)	<0.001	<0.001
Sodium mean mEq/L (SD)	139 (3.0)	137 (4.7)	139 (2.9)	<0.001	<0.001
BUN mean mg/dL (SD)	16 (9.5)	26 (20)	16 (9.0)	<0.001	0.66
Creatinine mean mg/dL (SD)	0.96 (0.7)	1.4 (1.4)	0.95 (0.68)	<0.001	0.43
Albumin mean g/dL (SD)	4.0 (0.58)	3.2 (0.8)	4.0 (0.55)	<0.001	<0.001
WBC count mean $\times 10^3/\mu L$ (SD)	8.1 (3.6)	11 (6.6)	8.1 (3.5)	<0.001	0.55
Haematocrit mean % (SD)	40 (5.3)	35 (7.1)	40 (5.2)	<0.001	<0.001
Operation time mean minutes (SD)	118 (96)	158 (138)	117 (95)	<0.001	0.34
LOS mean days (SD)	1.9 (9.1)	<0.001 (56)*	2.4 (4.0)	<0.001	<0.001

**Categorical variables**	**Total (n=963,565)**	**Excluded (n=18,060)**	**Included (n=945,505)**	**p-value**	**Cramér's V**	**χ^2 statistic**
**Self-designated Sex**				**<0.001**	**0.03**	841
Female	558,157 (58)	8,567 (47)	549,590 (58)			
Male	405,067 (42)	9,493 (53)	395,574 (42)			
Non-binary	337 (0.035)	0 (0.0)	337 (0.036)			
Intersex	4 (<0.001)	0 (0.0)	4 (<0.001)			
**Self-designated Race**				**<0.001**	**0.017**	219
Black or African American	95,921 (10.0)	2,073 (11)	93,848 (9.9)			
White	576,189 (60)	8,796 (49)	567,393 (60)			
Unknown	233,451 (24)	6,274 (35)	227,177 (24)			
American Indian or Alaska Native	7,111 (0.74)	126 (0.7)	6,985 (0.74)			
Multiple Races	6,697 (0.7)	104 (0.58)	6,593 (0.7)			
Asian	36,991 (3.8)	597 (3.3)	36,394 (3.8)			
Hispanic or Latino	2,156 (0.22)	35 (0.19)	2,121 (0.22)			
Native Hawaiian or Other Pacific Islander	3,877 (0.4)	42 (0.23)	3,835 (0.41)			
Middle Eastern or North African	1,172 (0.12)	13 (0.072)	1,159 (0.12)			
**Self-Designated Ethnicity**						
Hispanic	105,893 (11)	1,276 (7.1)	104,617 (11)	<0.001	0.013	124
**Diabetes**				**<0.001**	**0.057**	3114
No	805,455 (84)	13,165 (73)	792,290 (84)			
Non-insulin	111,255 (12)	2,466 (14)	108,789 (12)			
Insulin	46,855 (4.9)	2,429 (13)	44,426 (4.7)			
Current Smoker	114,477 (12)	3,319 (18)	111,158 (12)	<0.001	0.028	741
**Functional Status**				**<0.001**	**0.14**	19464
Independent	921,270 (96)	14,470 (80)	906,800 (96)			
Partially Dependent	18,853 (2.0)	2,395 (13)	16,458 (1.7)			
Totally Dependent	2,976 (0.31)	667 (3.7)	2,309 (0.24)			
Unknown	20,466 (2.1)	528 (2.9)	19,938 (2.1)			
Ventilator	2,105 (0.22)	1,297 (7.2)	808 (0.085)	<0.001	0.21	40905
COPD	35,842 (3.7)	2,160 (12)	33,682 (3.6)	<0.001	0.06	3487
Ascites	5,306 (0.55)	1,112 (6.2)	4,194 (0.44)	<0.001	0.1	10554
CHF	36,264 (3.8)	3,099 (17)	33,165 (3.5)	<0.001	0.097	9115
Hypertension	414,788 (43)	10,885 (60)	403,903 (43)	<0.001	0.048	2226
Renal Failure	1,776 (0.18)	600 (3.3)	1,176 (0.12)	<0.001	0.1	9833
Dialysis	7,498 (0.78)	1,221 (6.8)	6,277 (0.66)	<0.001	0.094	8524
Disseminated Cancer	20,960 (2.2)	1,798 (10.0)	19,162 (2.0)	<0.001	0.074	5232
Corticosteroid use	44,106 (4.6)	1,826 (10)	42,280 (4.5)	<0.001	0.037	1289
Bleeding Disorder	33,452 (3.5)	2,798 (15)	30,654 (3.2)	<0.001	0.091	7933
Blood Transfusion	7,430 (0.77)	1,740 (9.6)	5,690 (0.6)	<0.001	0.14	18885
Sepsis	62,029 (6.4)	6,775 (38)	55,254 (5.8)	<0.001	0.17	29505
Preop COVID	1,837 (0.19)	234 (1.3)	1,603 (0.17)	<0.001	0.035	1175

Oxygen support	19,031 (2.0)	3,644 (20)	15,387 (1.6)	<0.001	0.18	31487
**Case type**				**<0.001**	**0.17**	26368
Elective	786,346 (82)	6,861 (38)	779,485 (82)			
Emergent	77,432 (8.0)	6,454 (36)	70,978 (7.5)			
Urgent	99,787 (10)	4,745 (26)	95,042 (10)			
**Home Support**				**0.37**	**0.0024**	0.79
Unknown	12,032 (1.2)	211 (1.2)	11,821 (1.3)			
Lives at home with others	103,639 (11)	3,911 (22)	99,728 (11)			
Lives alone at home	34,681 (3.6)	1,346 (7.5)	33,335 (3.5)			
**Fall in past 6 months**				**<0.001**	**0.11**	1665
Yes	27,976 (2.9)	2,502 (14)	25,474 (2.7)			
Unknown	14,391 (1.5)	623 (3.4)	13,768 (1.5)			
Dementia	14,138 (1.5)	1,658 (9.2)	12,480 (1.3)	**<0.001**	0.11	1959
**Surgical specialty**				**<0.001**	**0.084**	6856
Plastics	31,027 (3.2)	124 (0.69)	30,903 (3.3)			
Gynaecology	98,844 (10)	242 (1.3)	98,602 (10)			
General Surgery	400,367 (42)	9,540 (53)	390,827 (41)			
Otolaryngology	23,406 (2.4)	203 (1.1)	23,203 (2.5)			
Urology	63,852 (6.6)	546 (3.0)	63,306 (6.7)			
Obstetrics	19,119 (2.0)	87 (0.48)	19,032 (2.0)			
Neurosurgery	49,880 (5.2)	1,494 (8.3)	48,386 (5.1)			
Vascular	28,783 (3.0)	1,676 (9.3)	27,107 (2.9)			
Thoracic	13,138 (1.4)	384 (2.1)	12,754 (1.3)			
Orthopaedics	231,270 (24)	3,459 (19)	227,811 (24)			
Cardiac surgery	3,813 (0.4)	302 (1.7)	3,511 (0.37)			
Interventional radiology	66 (0.0)	3 (0.0)	63 (0.0)			
**Anaesthesia**				**<0.001**	**0.023**	506
General	835,733 (87)	16,639 (92)	819,094 (87)			
Epidural	5,196 (0.54)	42 (0.23)	5,154 (0.55)			
MAC/IV sedation	51,652 (5.4)	682 (3.8)	50,970 (5.4)			
Regional	5,987 (0.62)	83 (0.46)	5,904 (0.62)			
Spinal	63,567 (6.6)	582 (3.2)	62,985 (6.7)			
Local	970 (0.1)	17 (0.094)	953 (0.1)			
Unknown	418 (0.043)	15 (0.083)	403 (0.043)			
**Setting**				**<0.001**	**0.13**	16767
Outpatient	500,143 (52)	761 (4.2)	499,382 (53)			
Inpatient	463,422 (48)	17,299 (96)	446,123 (47)			
Major complication	94,002 (9.8)	12,460 (69)	81,542 (8.6)	<0.001	0.28	73350
Delirium	12,192 (1.3)	2,637 (15)	9,555 (1.0)	<0.001	0.24	9100
Home discharge	897,311 (93)	2,958 (16)	894,353 (95)	<0.001	0.42	169283
Home discharge (with services)	34,474 (3.6)	522 (2.9)	33,952 (3.6)	<0.001	0.058	423
End-of-life care	6,713 (0.7)	5,541 (31)	1,172 (0.12)	<0.001	0.5	239136
Postop COVID	4,060 (0.42)	313 (1.7)	3,747 (0.4)	<0.001	0.028	752
Functional decline	42,248 (4.4)	1,422 (7.9)	40,826 (4.3)	<0.001	0.1	1486

BMI, Body Mass Index; BUN, Blood Urea Nitrogen; CHF, Congestive Heart Failure; COPD, Chronic Obstructive Pulmonary Disease; LOS, length of hospital stay; MAC/IV, Monitored Anesthesia Care/Intravenous sedation; WBC, White Blood Cell count. * due to LOS-based exclusion criteria
